# Enhanced inflammation and attenuated tumor suppressor pathways are associated with oncogene‐induced lung tumors in aged mice

**DOI:** 10.1111/acel.12691

**Published:** 2017-10-18

**Authors:** Neha Parikh, Ryan L. Shuck, Mihai Gagea, Lanlan Shen, Lawrence A. Donehower

**Affiliations:** ^1^ Department of Molecular Virology and Microbiology Baylor College of Medicine Houston TX 77030 USA; ^2^ Department of Veterinary Medicine and Surgery UT MD Anderson Cancer Center Houston TX 77030 USA; ^3^ Children's Nutrition Research Center Houston TX 77030 USA

**Keywords:** aging, cancer, inflammation, Kras, p53, tumor microenvironment

## Abstract

Aging is often accompanied by a dramatic increase in cancer susceptibility. To gain insights into how aging affects tumor susceptibility, we generated a conditional mouse model in which oncogenic *Kras* ^*G12D*^ was activated specifically in lungs of young (3–5 months) and old (19–24 months) mice. Activation of *Kras* ^*G12D*^ in old mice resulted in shorter survival and development of higher‐grade lung tumors. Six weeks after *Kras* ^*G12D*^ activation, old lung tissues contained higher numbers of adenomas than their young tissue counterparts. Lung tumors in old mice displayed higher proliferation rates, as well as attenuated DNA damage and p53 tumor suppressor responses. Gene expression comparison of lung tumors from young and old mice revealed upregulation of extracellular matrix‐related genes in young tumors, indicative of a robust cancer‐associated fibroblast response. In old tumors, numerous inflammation‐related genes such as *Ccl7*,*IL‐1*β, *Cxcr6,* and *IL‐15ra* were consistently upregulated. Increased numbers of immune cells were localized around the periphery of lung adenomas from old mice. Our experiments indicate that more aggressive lung tumor formation in older *Kras* ^*G12D*^ mice may be in part the result of subdued tumor suppressor and DNA damage responses, an enhanced inflammatory milieu, and a more accommodating tissue microenvironment.

## Introduction

Cancer is an age‐associated disease. While cancer occurs at all ages, overall cancer risk rises exponentially with age. Humans exhibit rapidly increasing cancer rates starting in the sixth decade of life (DePinho, 2000; Campisi, 2003). In the laboratory, most inbred mouse strains with a maximum lifespan of 24–36 months begin to show exponential increases in cancer risk after 18 months. While many mouse cancer models and a modest number of mouse aging models have been developed and characterized, the direct relationship between cancer and aging has rarely been explored in either cancer or aging models. Given the complexities of both processes in mammalian organisms, devising definitive experiments to understand specific causal mechanisms in the aging–cancer relationship has been a major challenge.

Multiple theories have been proposed to explain the age dependence of somatically arising cancers. As early as the 1950s, it was hypothesized that cancers were a product of a multistep genetic lesions arising somatically in specific cells (Nordling, 1953; Armitage & Doll, 1954). Cancer genome sequencing has confirmed that the vast majority of individual cancers display mutations in multiple genes known functionally to be cancer driver genes (Lawrence *et al*., 2013). Thus, the age dependence of cancer is likely driven in large measure by the statistical likelihood over time that a given set of complementary driver mutations occurs within a single normal cell that transforms it into a malignant cancer cell. Recently, the multistage carcinogenesis model has been further refined by Tomasetti & Vogelstein (2015) to show that lifetime risk of a cancer type correlates with total number of normal stem/progenitor cell divisions that maintain homeostasis within the tissue of origin. Two‐thirds of cancers were estimated to be caused by stochastic replication errors in self‐renewing cells within a tissue.

Despite the elegance of the above model, it may be incomplete because of its focus on cell autonomous genetic events in the evolving cancer cell. Different cancer types arise at similar rates even though their stem cell pools may have very different sizes, division rates, and mutational spectra. As proposed by Rozhok & DeGregori (2015), noncell autonomous processes such as age‐dependent tissue microenvironmental changes may play an important role in accelerating cancer development. The aged tissue microenvironment may provide a selective advantage in enhancing the oncogenic effects of mutated cancer driver genes compared to the youthful tissue microenvironment. Campisi and collaborators have demonstrated that senescent fibroblasts were more effective than young fibroblasts in stimulating premalignant and malignant epithelial cells to proliferate *in vitro* and to form tumors in mice (Krtolica *et al*., 2001). This senescence‐dependent tumorigenic effect was driven in part by secretion of an array of growth stimulatory inflammatory cytokines (Coppe *et al*., 2010). Indeed, chronic low‐grade inflammation, also known as ‘inflammaging’, is frequently seen in old age and is strongly correlated with mortality (Lopez‐Otin *et al*., 2013; Morrisette‐Thomas *et al*., 2014). Many genes associated with inflammation and immune responses are shown to be increased in aged tissues from humans and rodents (de Magalhaes *et al*., 2009). Thus, as more stromal cells undergo senescence in the aging tumor microenvironment, the activation of the senescence‐associated secretory phenotype (SASP) may stimulate progression of oncogenically activated premalignant cells in the vicinity.

To examine potential mechanisms of age‐associated tumorigenesis in an intact animal, we used a genetically engineered conditional mouse model of lung cancer. A Cre recombinase‐inducible mutant *Kras* ^*G12D*^ allele was specifically activated in lungs by inhalation of Cre adenovirus (DuPage *et al*., 2009) to study age‐associated differences in lung tumor formation. *KRAS* mutations represent potent oncogenic driver events that occur in 35% of lung cancers and 30% of all human cancers (Wilson & Tolias, 2016). We compared the tumorigenic effects of *Kras* ^*G12D*^ activation in young (3–5 months) and old mice (19–24 months). We hypothesized that if tumors showed more rapid development and progression in the old *Kras*‐induced mice, this would be more supportive of models emphasizing cell‐nonautonomous mechanisms rather than strict cell autonomous mutational mechanisms. Genomic mutation loads in mice increase less than twofold between 4 and 20 months of age (Vijg *et al*., 2005), so a strict mutational model would not be expected to produce dramatic differences in tumorigenesis between young and old mice. In fact, we did observe significantly accelerated tumorigenesis and the appearance of higher‐grade lung tumors in old mice. Further analyses to identify molecular mechanisms showed attenuated tumor suppressor pathway responses and DNA damage responses in lung tumors from old mice relative to their young counterparts. Evidence of differential noncell autonomous effects included likely enhanced activation of fibroblast‐secreted extracellular matrix (ECM) components in young tumors and immune cell secretion of inflammatory cytokines in older tumors. We propose that the combination of cell intrinsic attenuated tumor suppressor/DNA damage responses and cell extrinsic tumor microenvironment fibroblast functions and immune cell inflammatory cytokine effects play significant roles in the enhanced tumorigenesis observed in the older *Kras* ^*G12D*^ mice.

## Results

### Establishing the inducible bi‐allelic *Kras* ^*G12D*^ *; LacZ* model and confirmation of comparable *Kras* ^*G12D*^ activation in young and old mice

**Figure 1:**
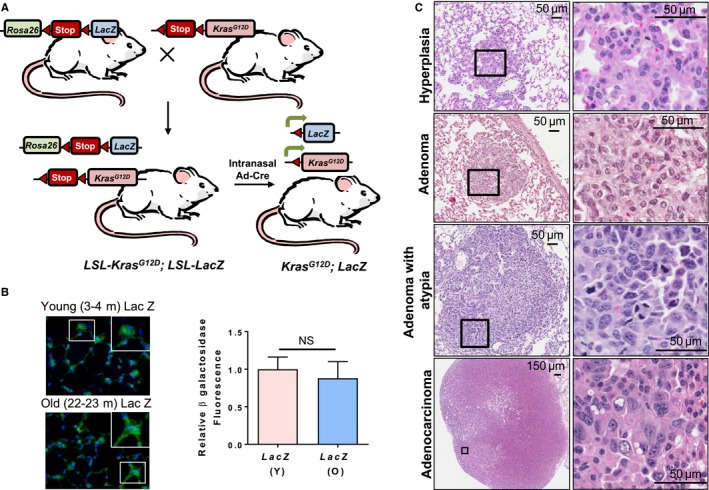
Intranasal instillation of Cre adenovirus leads to lacZ expression and lung tumors in *LSL‐Kras* ^*G12D*^; *LSL‐Rosa26‐LacZ* mice. (A) Schematic representation of the generation of *Kras* ^*G12D*^ *; LacZ* mice and Cre adenovirus‐mediated induction of *Kras* ^*G12D*^ and *LacZ* encoding β–galactosidase expression. (B) β‐Galactosidase staining assessed by immunofluorescence (green) in lungs from young and old monoallelic mice harvested 6 weeks post‐Cre adenovirus instillation. Nuclei are stained with DAPI (blue). Inset shows magnified images of cells expressing β‐galactosidase. Panel at right indicates relative β‐galactosidase staining in young and old *LacZ* mice 6 weeks after Cre adenovirus instillation. (C) Histopathological classification of lung tumors and hyperplasia based on H&E‐stained lung sections in mice six weeks after Cre adenovirus (1.25 × 10^6^ PFU) instillation shows bronchioloalveolar hyperplasia, adenoma, adenoma with atypia, and adenocarcinoma. Adenoma with atypia was considered a subcategory of adenomas that contained more than 10 atypical cells with pleomorphic nuclei, but did not have typical adenocarcinoma features. Square insets in the left‐side panels are shown at higher magnification in the right panels.

To achieve expression of oncogenic Kras^G12D^ along with a reporter gene, we generated bi‐allelic *Kras* ^*G12D*^; *LacZ* mice by crossing *Lox‐Stop‐Lox‐Kras* ^*G12D*^ (*LSL‐Kras* ^*G12D*^) mice with *Rosa26‐LSL LacZ* mice (Fig. 1A). In the resulting bi‐allelic mice, both the *Kras* ^*G12D*^ and *Rosa26‐LacZ* alleles are preceded by a *LoxP*‐flanked stop cassette that prevents transcription of the downstream gene. When Cre recombinase is delivered via adenovirus expressing Cre, it binds to the *LoxP* recombination sites and deletes the *LSL* cassette, activating transcription of *Kras* ^*G12D*^ and *LacZ*. Intranasal administration of Cre adenovirus in young (3–5 months) and old (19–24 months) mice would result in deletion of the *LSL* cassettes and expression of *LacZ* and *Kras* ^*G12D*^ in lungs of bi‐allelic *Kras* ^*G12D*^; *LacZ* mice (Fig. 1A). For simplicity, the bi‐allelic *Kras* ^*G12D*^; *LacZ* mice will henceforth be referred to as *Kras* ^*G12D*^ mice. *LacZ* reporter gene expression was used to address variation in rates of activation by Cre adenovirus, as the nature of intranasal instillation entails some variability in the delivery of Cre adenovirus to individual lungs. Immunofluorescence analysis for β‐galactosidase (β‐gal) in lung sections from young (4 months) and old (24 months) *LacZ* control mice averaged roughly similar levels of staining at 6 weeks postinstillation (Fig. 1B and Fig. S1A). Western blot analysis showed increased pErk1/2 expression indicating activated KRas^G12D^ signaling in lungs harvested 6 weeks postinstillation from *Kras* ^*G12D*^ mice (young and old) (Fig. S1B). Despite some mouse to mouse variability, there were no remarkable differences in LacZ expression between young and old mice at 6 weeks post‐Cre adenovirus. Also, increased detection of β‐gal in bi‐allelic mice relative to *LacZ* controls indicates preferential proliferation of adenovirus‐transduced Kras^G12D^ and β‐gal expressing cells (Fig. S1B). LSL recombination was compared using genomic DNA and primers for the recombined *LacZ* gene in control lungs harvested 6 weeks post‐Cre adenovirus (Fig. S1C). Despite some variability, no significant age‐related differences were found in the LSL recombination for LacZ. Thus, LSL recombination (Fig. S1) and β‐gal expression (Fig. 1B and Fig. S1A,B) did not display significant differences between young and old *Kras* ^*G12D*^ mice, indicating similar fractions of lung cells with activated *Kras* ^*G12D*^ at 6 weeks post‐Cre. Lungs harvested 2 weeks post‐Cre were also subjected to Western blot analyses for β‐gal. Lungs from *Kras* ^*G12D*^ mice expressed more β‐gal than matched *LacZ* control lungs (Fig. S2A). Further, β‐gal expression in lungs was assessed by X‐gal staining. In agreement with immunoblot data, *Kras* ^*G12D*^ mice showed enhanced X‐gal staining relative to control *LacZ* mice due to hyperproliferation induced by activated *Kras* ^*G12D*^ (Fig. S2B). Similar to our previous Western blot data, β‐gal expression in old mice is equivalent or slightly lower than young mice at 2 weeks post‐Cre adenovirus.

Lungs from Cre adenovirus‐instilled *Kras* ^*G12D*^ mice showed hyperproliferation leading to different grades of lung lesions previously reported in mouse lung tumor models (Jackson *et al*., 2001; DuPage *et al*., 2009) such as bronchioloalveolar hyperplasia, adenoma, and adenocarcinoma (Fig. 1C, Methods S1). In this study, lung adenoma with atypia is a subclassification of lung adenoma that consisted of adenoma with additional presence of clusters of 10 or more atypical cells that had enlarged and pleomorphic nuclei, but no other features of malignancy. Unless explicitly mentioned, adenoma in the text refers to all adenomas (including adenoma with atypia).

### Old *Kras* ^*G12D*^ mice show shorter survival and more advanced end‐stage lung tumors

**Figure 2:**
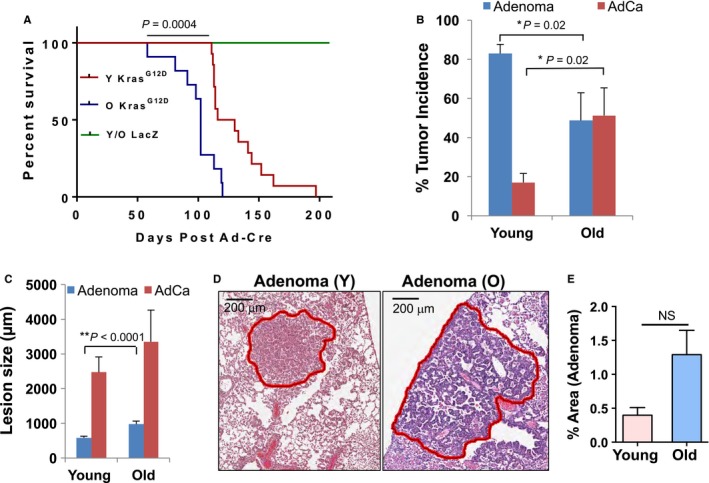
Old mice show shorter survival and more aggressive tumors at the end stage of tumor analyses. (A) Old *Kras* ^*G12D*^ mice (19–20 months, *n* = 11) have lower cumulative survival than young *Kras* ^*G12D*^ mice (3–5 months, *n* = 14) as measured by log‐rank survival analysis. (B) The incidence of lung adenoma and adenocarcinoma in young (3–5 months, *n* = 8) and old *Kras* ^*G12D*^ mice (19–20 months, *n* = 7) was assessed histologically on H&E‐stained median lung sections from each mouse. Based on histological analysis, the incidence for adenoma and adenocarcinoma (AdCa) was calculated as the percentage of adenoma or adenocarcinoma of total number of lung tumors observed per lung section and per mouse (*n* = 52 lung tumors in young and *n* = 37 lung tumors in old mice). (C) The size of tumors was analyzed and compared between young and old *Kras* ^*G12D*^ mice by measuring the largest diameter of each lung tumor/lesion using Aperio ImageScope software. Data from 44 adenoma lesions and nine adenocarcinomas from eight young mice, 26 adenoma lesions and 11 adenocarcinoma lesions from 7 old mice are plotted. (D) Representative digital images of scanned H&E lung sections used for morphometric analysis of adenoma from young (Y) and old (O) *Kras* ^*G12D*^ mice. (E) Percent tumor area (including adenoma and adenocarcinoma) with respect to total lung surface area was obtained using Aperio ImageScope software and is presented in the plotted diagram for old and young *Kras* ^*G12D*^ mice in the tumor latency study.

To compare the long‐term effect of age on tumor formation and progression, young (3–5 months) and old (19–20 months) *Kras* ^*G12D*^ mice were subjected to Cre adenovirus instillation and tumor latency analysis. Comparative analysis of tumor latency showed old activated *Kras* ^*G12D*^ mice with significantly reduced survival as compared to young activated *Kras* ^*G12D*^ mice (*P* = 0.0004) (Fig. 2A, Data S1). Both young and old age‐matched control *LacZ* mice showed no mortality for the duration of the study (200 days). Lung tumors harvested terminally in the latency study were assessed histopathologically and classified as adenoma or adenocarcinoma on hematoxylin and eosin (H&E)‐stained lung sections. These lungs also displayed areas of severe hyperplasia but only clearly defined lesions of adenoma and adenocarcinoma were included for this detailed analysis. Histopathological analysis of lung tumors from young *Kras* ^*G12D*^ mice revealed that ~ 80% of lung tumors were adenoma lesions and 20% of tumors were adenocarcinoma lesions. In contrast, a significantly higher proportion of old *Kras* ^*G12D*^ mice lung tumors were adenocarcinoma (~ 50%) and a lower proportion of tumors were adenoma (~ 50%) (Fig. 2B). In addition, tumor area evaluation indicated that old *Kras* ^*G12D*^ mice displayed significantly larger adenoma lesions than adenomas from young *Kras* ^*G12D*^ mice (Fig. 2C,D). There were no significant differences observed in total lung tumor area (Fig. 2E) and number of lung tumors (adenoma plus adenocarcinoma) between young and old *Kras* ^*G12D*^ mice (not shown) indicating comparable tumor burden at the moribund stage. Collectively, these data indicate slower growing and lower grade tumors arising in young mice associated with longer survival post‐Cre induction. No distant metastases were observed in the *Kras* ^*G12D*^ mice.

### Short‐term studies reveal old *Kras* ^*G12D*^ mice with more aggressive tumors

**Figure 3:**
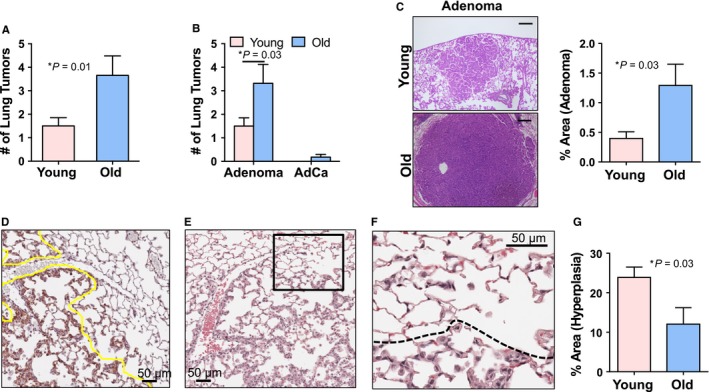
Old *Kras* ^*G12D*^ mice develop more aggressive tumors than young *Kras* ^*G12D*^ mice 6 weeks post‐Cre adenovirus instillation. (A) Old *Kras* ^*G12D*^ mice (22–24 months, *n* = 19) show significantly increased tumor number compared to young *Kras* ^*G12D*^ mice (3–5 months, *n* = 20) 6 weeks post‐Cre adenovirus instillation. Average numbers of adenoma and adenocarcinoma lesions observed per each H&E‐stained lung section and per mouse are plotted. (B) Old *Kras* ^*G12D*^ mice develop significantly higher numbers of adenoma lesions 6 weeks post‐Cre adenovirus instillation. Adenocarcinomas were not observed in young *Kras* ^*G12D*^ mice, but only in old *Kras* ^*G12D*^ mice. Data from 77 tumors in 19 old mice and 30 tumors from 20 young mice are plotted. (C) Old *Kras* ^*G12D*^ mice develop larger adenoma lesions than young *Kras* ^*G12D*^ mice. Left panels: image of H&E‐stained lung sections showing representative adenomas from young (Y) and old (O) *Kras* ^*G12D*^ mice. Scale bar: 200 μm. Right panel: Percent of adenoma area with respect to total lung surface area was analyzed on H&E‐stained median lung sections in old (*n* = 15) and young (*n* = 13) *Kras* ^*G12D*^ mice. (D) Image of lung section immunostained for keratin 8 and counter stained with hematoxylin shows hyperplastic epithelial cells staining intensely with keratin 8 antibody as delineated under the yellow line. (E and F) H&E‐stained section from the same mouse shows alveoli with enlarged hyperplastic epithelial cells distinguished from adjacent normal alveolar pneumocytes. Inset in (E) shown at larger magnification in (F). Dashed line in the enlarged inset demarcates alveoli with the hyperplastic epithelial cells from normal alveoli. (G) Percent of hyperplastic area with respect to total lung surface area was significantly higher in young (*n* = 8) compared to old (*n *= 8) *Kras* ^*G12D*^ mice. Areas of bronchioloalveolar hyperplasia were analyzed based on H&E‐stained and Keratin 8 immunostained lung sections using Aperio ImageScope software.

To test whether the more aggressive lung tumor phenotype in old *Kras* ^*G12D*^ mice appears at earlier time points after Kras activation, lungs of young (3–5 months) and old (22–24 months) activated *Kras* ^*G12D*^ mice were examined histopathologically 6 weeks after intranasal instillation with Cre adenovirus. The average number of tumors (adenoma plus adenocarcinoma) observed in H&E‐stained sections in old *Kras* ^*G12D*^ mice significantly exceeded the number of tumors observed in lung sections from young *Kras* ^*G12D*^ mice (Fig. 3A). Further analysis of lung lesions revealed much higher number of adenomas in old *Kras* ^*G12D*^ mice as compared to young *Kras* ^*G12D*^ mice (Fig. 3B). Subclassification of adenomas into adenoma with no atypia and adenoma with atypia revealed significantly higher number of adenoma with atypia in old *Kras* ^*G12D*^ mice (>Fig. S3). Similarly, adenocarcinoma was observed in 3/19 old *Kras* ^*G12D*^ mice but not in young *Kras* ^*G12D*^ mice (Fig. 3B). Comparative analysis of areas of all adenoma lesions revealed significantly larger adenoma lesions in old *Kras* ^*G12D*^ mice than in young *Kras* ^*G12D*^ mice (Fig. 3C). Hyperplasia was also compared in lungs from young and old mice. The hyperplastic epithelial cells were enlarged and stained intensely with antibody for keratin 8 (Fig. 3D). Closer examination of H&E‐stained sections of lungs showed that these mildly hyperplastic areas of lung have enlarged/hypertrophic alveolar epithelial cells with increased cytoplasm and larger nuclei than normal (Fig. 3E,F). The morphometric quantification of hyperplastic areas distinguished by intense keratin 8 and H&E staining revealed a higher percentage of the total area of bronchioloalveolar hyperplastic regions in young *Kras* ^*G12D*^ mice than in old *Kras* ^*G12D*^ mice (Fig. 3G), in contrast with observations of more advanced tumor types.

### Higher proliferative index observed in old *Kras* ^*G12D*^ tumors

**Figure 4:**
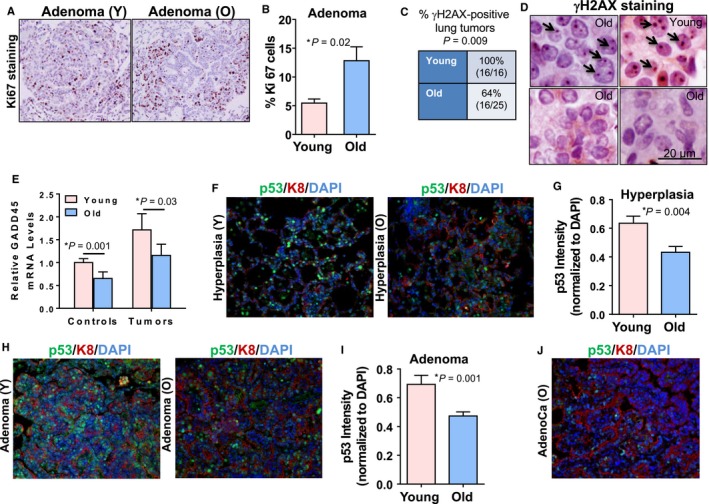
Tumors from old *Kras* ^*G12D*^ mice show increased proliferation, attenuated DNA damage responses, and reduced p53 tumor suppressor responses. (A) Images showing increased Ki67 immunostaining in a lung adenoma of an old *Kras* ^*G12D*^ mouse (O, 22–24 months) compared to an adenoma in a young *Kras* ^*G12D*^ mouse (Y, 3–5 months) 6 weeks post‐Cre adenovirus. (B) Quantitation of Ki67‐positive cells in lung tumors from young (13 lung adenomas from six mice) and old (20 lung adenomas from six mice) *Kras* ^*G12D*^ mice shows an increased proliferation index in adenomas from old mice. (C) Significantly reduced percentages of lung tumors are positive for γH2AX foci in old *Kras* ^*G12D*^ mice. Adenoma and adenocarcinoma lung lesions were categorized as positive for γH2AX foci based on the staining (reddish brown foci) in > 10% of cells. Table shows data from eight young (16 lesions) and nine old (24 lesions) mice. (D) Representative images of lung tumor positive (upper panels) and negative (lower panels) for γH2AX staining are shown. Arrows indicate individual γH2AX foci. Lung sections were counterstained with hematoxylin. (E) qPCR for Gadd45a mRNA showed decreased expression in normal lung tissue and lung tumors from old mice. LacZ control lungs from young (3–5 m, *n* = 8), and old mice (22–24 m, *n* = 5), and lung lesions from young (3–5 m, *n* = 9) and old (22–24 m, *n *= 5) *Kras* ^*G12D*^ mice were examined. (F) Reduced p53 immunostaining (green) is observed in lung hyperplasia from old (O) mice. Blue represents DAPI‐stained nuclei, and red represents keratin 8 (K8)‐stained epithelial cells. (G) Quantitation of p53 intensity overlapping with nuclei and normalized to DAPI intensity in hyperplastic areas from young (29 hyperplastic lesions from five mice) and old (22 hyperplastic lesions from five mice) *Kras* ^*G12D*^ mice indicate reduced p53 protein in old hyperplasias. Combined data from two independent experiments are plotted. (H) Reduced p53 immunostaining (green) is observed in old *Kras* ^*G12D*^‐induced mouse lung adenomas. Representative sections are shown. (I) Quantitation of p53 intensity in lung adenomas from young (nine adenoma lesions from four mice) and old (16 adenoma lesions from five mice) animals as described in panel G indicates an attenuated p53 response in old lung adenomas. Corresponding H&E‐stained lung sections were used as a reference to confirm lesions as lung adenoma. (J) Representative image for adenocarcinoma derived from old *Kras* ^*G12D*^ mouse immunostained for p53 (green) and keratin 8 (red) and counterstained with DAPI (blue) shows characteristic loss of p53 protein.

The incidence of more aggressive lung tumors in old *Kras* ^*G12D*^ mice led us to explore the molecular mechanisms responsible for phenotype differences. For all subsequent analyses, lungs from young (3–5 months) and old (22–24 months) *Kras* ^*G12D*^ mice were harvested 6 weeks post‐*Kras* ^*G12D*^ activation. Proliferation marker Ki67 staining of lung tumors showed increased numbers of positively stained cells in adenomas from old *Kras* ^*G12D*^ mice. Quantitative analysis of Ki67‐stained nuclei showed higher proliferative indexes in lung adenoma lesions from old *Kras* ^*G12D*^ mice as compared to young *Kras* ^*G12D*^ mice (Fig. 4A,B). However, we did not observe significant differences in Ki67 staining in hyperplastic tissues from young and old *Kras* ^*G12D*^ mice (Fig. S4A).

### The DNA damage response is attenuated in old *Kras* ^*G12D*^ mice

The DNA damage response in premalignant tumors acts as a barrier to cancer progression following oncogene‐induced replication stress and DNA double‐strand breaks associated with cell proliferation (Halazonetis *et al*., 2008). We investigated whether the DNA damage response differs in young and old mice with activated KRas^G12D^ signaling. γH2AX is a critical DNA damage repair protein activated in premalignant lesions that localizes to DNA strand breaks resulting from replication stress (Kinner *et al*., 2008). In the six‐week study, all of the 16 adenoma lesions analyzed from young *Kras* ^*G12D*^ mice (*n* = 8) were positive for γH2AX staining, whereas 64% (16/25) of the adenoma lesions from old *Kras* ^*G12D*^ mice (*n* = 9) were positive for γH2AX staining (Fig. 4C,D). The γH2AX‐positive tumors had 80–90% cells with γH2AX foci irrespective of age (Fig. S4B), and γH2AX‐negative tumors had at least 90% of cells with no γH2AX foci (not shown). Transcript levels of DNA damage‐induced tumor suppressor Gadd45a were significantly downregulated in healthy lungs and tumors from old mice as compared to young counterparts (Fig. 4E) indicating a blunted DNA damage response with increased age.

### Tumor suppressor responses are less robust in premalignant lesions from old *Kras* ^*G12D*^ mice

Oncogene‐induced cell proliferation activates tumor suppressor responses that deter further tumor progression. Hence, we sought to examine the effects of age on tumor suppressor responses by p53, p19^Arf^, p16^Ink4a^, p27^Kip1^, and p21^Cip1^. Lungs from old and young *Kras* ^*G12D*^ mice harvested 6 weeks after Cre adenovirus instillation were stained for tumor suppressor p53 protein (green). Keratin 8 (red) was used to mark hyperplastic epithelial lesions in lungs. Hyperplastic areas from young *Kras* ^*G12D*^ mice showed more intense and widespread p53 induction (Fig. 4F, first panel) relative to hyperplastic lung areas from old *Kras* ^*G12D*^ mice (Fig. 4F, second panel). Nuclear p53 intensity was quantified for multiple hyperplastic lung areas, and old *Kras* ^*G12D*^ mice showed significantly reduced p53 staining in hyperplasias compared to their young mouse counterparts (Fig. 4G). Similarly, adenomas from young and old *Kras* ^*G12D*^ mice were assessed for p53 upregulation. Overall, there was less nuclear p53 expression in adenomas from old *Kras* ^*G12D*^ mice (Fig. 4H,I). Lung adenocarcinomas from old *Kras* ^*G12D*^ mice showed minimal p53 staining consistent with the advanced nature of those tumors (Fig. 4J). These data indicate that aging inhibits timely and efficient onset of the p53 tumor suppressor response in a premalignant context.

We also assessed the mRNA levels of p19^Arf^ and p16^Ink4a^ in lung lesions from young and old *Kras* ^*G12D*^ mice (Fig. S4C,D). As previously reported for these tumor suppressors (Krishnamurthy *et al*., 2004), normal lung tissues of old mice showed significantly higher levels of p19^Arf^ and p16^Ink4a^ RNA compared to young mice. p19^Arf^ and p16^Ink4a^ RNA were significantly upregulated in lung lesions from young mice, indicating an intact p16/p19 tumor suppressor response. However, further increases in p19^Arf^ and p16^Ink4a^ RNA were not observed in old lung lesions relative to already elevated levels of these RNAs observed in age‐matched normal lung tissue, suggesting the absence of an induction of significant tumor suppressor response to oncogenic stimulus in older mice. Similarly, p21^Cip1^ mRNA levels were induced in lung tumors of young *Kras* ^*G12D*^ mice but not in lung tumors of old *Kras* ^*G12D*^ mice when compared to age‐matched controls (Fig. S4E). Finally, no significant differences were found between young and old *Kras* ^*G12D*^ mice lung adenomas for p27^Kip1^ protein staining (Fig. S4F,G).

### DNA methylation analyses reveal hypermethylation of the *Cadherin 13* promoter in lungs of old mice

Aberrant DNA methylation is common in cancer and aging (Sandoval & Esteller, 2012; Jung & Pfeifer, 2015). We examined global and site‐specific changes in DNA methylation in our lung cancer model. Global methylation levels assessed by quantitative bisulfite pyrosequencing of retrotransposons IAP and line‐1 showed no methylation changes in healthy lung tissue and lung tumors as a function of age (Fig. S5A,B). One tumor suppressor that is shown to be downregulated and hypermethylated in many cancers (including lung cancer) is cadherin 13 (*CDH13*). (Andreeva & Kutuzov, 2010; Xue *et al*., 2014). Based on microarray data from *Kras* ^*G12D*^ mice derived lung lesions, validation by qRT–PCR showed twofold downregulation of Cdh13 in healthy lung and lung lesions from old mice (Fig. S5C). Next, we examined epigenetic changes in the *Cdh13* promoter region in lungs from young and old mice. The *Cdh13* promoter was found to be significantly hypermethylated in old normal lungs relative to young normal lungs (Fig. S5D) and in lung premalignant lesions from old mice relative to their young lung lesion counterparts (Fig. S5E). Examination of the aging‐relevant *Cdkn2a* (encoding p16^Ink4a^) gene showed that its promoter was moderately more hypermethylated in normal lungs of old mice relative to young mice, but no significant differences in *Cdkn2a* promoter methylation were found in lung tumors from young and old *Kras* ^*G12D*^ mice (Fig. S5F).

### RNA expression analyses reveal changes in the tumor microenvironment of young and old *Kras* ^*G12D*^ mice

**Figure 5:**
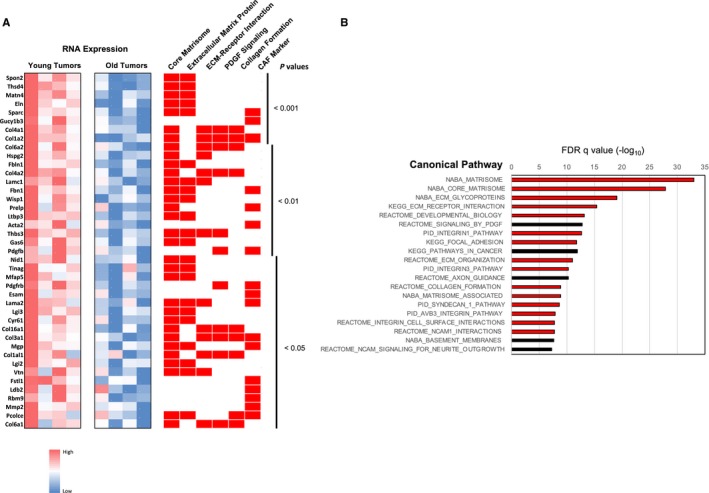
Lung adenomas from young *Kras* ^*G12D*^ mice show preferential upregulation of genes associated with extracellular matrix (ECM) and cancer‐associated fibroblasts (CAFs). (A) Microarray results comparing gene expression in four young and four old *Kras* ^*G12D*^ tumors presented as a heat map. Representative significantly upregulated genes with *P* values < 0.05 in young *Kras* ^*G12D*^ mice are shown at left and their relative expression indicated by the heat map. To the right of the heat map, red rectangles indicate canonical pathway associations for each gene. The right most category, ‘CAF Marker’, represent those genes consistently shown in the literature to be associated with upregulation in CAFs. *P* values to the right of the pathways indicate significance of young vs. old differential expression for each gene. (B) Genes overexpressed in young *Kras* ^*G12D*^ tumors show dramatic enrichment for ECM‐associated pathways by Gene Set Enrichment Analysis (GSEA). The top 20 most significantly enriched canonical pathways are shown based on GSEA of the list of the 359 significantly upregulated genes in young *Kras* ^*G12D*^ tumors relative to old *Kras* ^*G12D*^ tumors. Relative *P* values (−log_10_) are indicated above the graph. Red bars indicate pathways related to ECM structure and function.

Microarray expression analysis was performed on four young and four old *Kras* ^*G12D*^ adenomas 6 weeks after Kras^G12D^ activation. Statistical comparison of the young and old lesions revealed 359 genes significantly increased in RNA expression in young tumors relative to old tumors (Table S1, Fig. 5A). Gene Set Enrichment Analysis (GSEA) of the 359 genes showed a highly significant enrichment in canonical pathways related to the ECM and genes specific to cancer‐associated fibroblasts (CAFs) (Table S2, Fig. 5B). Numerous collagens and other ECM structural molecules such as Sparc, Lama2, and Fbn1 were significantly upregulated in the young tumors. Standard CAF marker genes such as alpha smooth muscle actin (Acta2), Pdgfb, Pdgfrb, and Mmp2 exhibited increased expression in the young lesions. Since gene expression patterns are derived from tumor lesions with mixed populations of cells, key CAF marker gene Acta2 expression was further examined using immunostaining. Antibody staining of young and old *Kras* ^*G12D*^ tumor sections for smooth muscle actin protein expression showed overall enhanced staining in young tumors relative to old tumors, although differences did not reach significance (Fig. S6). Given that most of these differentially regulated genes are primarily expressed in fibroblasts, these results suggest that the expression differences in the young and old lesions arise from the tumor‐associated stroma rather than the epithelial lesions themselves.

**Figure 6:**
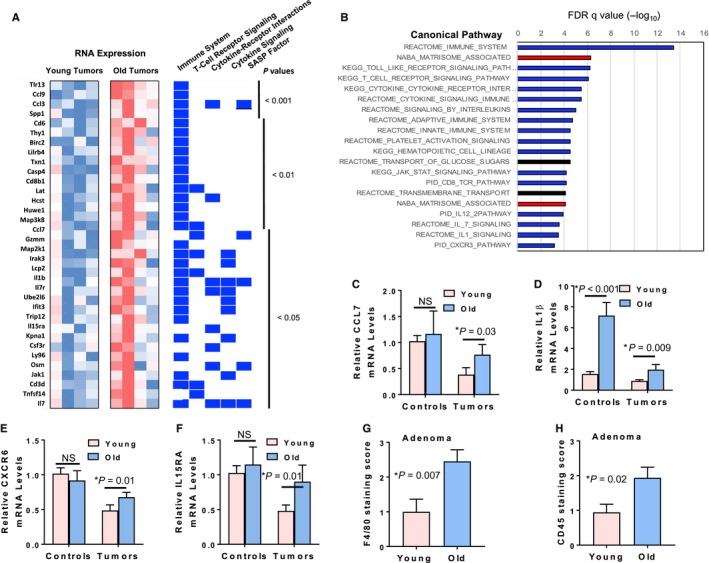
Lung adenomas from old *Kras* ^*G12D*^ mice show upregulation of genes affiliated with immune cells and inflammatory pathways. (A) Microarray results show enhanced expression of multiple inflammatory pathway genes in lung tumors from old *Kras* ^*G12D*^ mice. Representative upregulated genes in old *Kras* ^*G12D*^ tumors with *P* value < 0.05 were compared for relative expression in young and old tumors as shown in the heat map. Each gene was also assessed for association (indicated by blue rectangles) with immune cell and cytokine pathways using GSEA. The last category, ‘SASP Factor’, indicates those genes associated in the literature with the senescence‐associated secretory phenotype. (B) Genes overexpressed in old *Kras* ^*G12D*^ tumors show enrichment for immune cell and inflammatory pathways by GSEA. The top 20 most significantly enriched canonical pathways are shown based on GSEA of the 176 significantly upregulated genes in old *Kras* ^*G12D*^ tumors relative to young *Kras* ^*G12D*^ tumors. Relative *P* values (−log_10_) are indicated above the graph. Blue bars indicate immune cell/cytokine/inflammatory pathways, and red bars indicate extracellular matrix‐associated pathways. (C–F) qPCR validation shows significant upregulation of inflammation‐related genes Ccl7 (C), IL‐1 β (D), Cxcr6 (E) and IL‐15ra (F) in lung tumors from old *Kras* ^*G12D*^ mice (*n* = 5 for young and old mice). For control samples (*n* = 5 for young and old mice), only IL‐1‐β showed significant upregulation in old mice (D). (G–H) Immune cells cluster in close proximity to the lung tumors derived from old mice. Lung adenomas from young and old *Kras* ^*G12D*^ mice stained for macrophages (F4/80 antibody, G) and leukocytes (CD45 antibody, H) were visually assessed and scored on a scale of 0–4 by a pathologist based on the estimate of immune cells present within the tumor or within 100 μm of the tumor boundary. Twenty lung tumors from six old mice and 14 lung tumors from five young mice were examined for F4/80 staining. Twenty‐four lung tumors from five old mice and 18 lung tumors from six young mice were examined for CD45 staining.

Statistical analysis of RNA expression also revealed that 176 genes were significantly upregulated in the old adenomas (Table S3, Fig. 6A). GSEA on this gene set showed a dramatic enrichment of immune cell markers and cytokines and chemokines associated with inflammatory pathway gene expression, including IL‐1β, IL‐7, Ccl3, Ccl7, Ccl9, and Osm (Table S4, Fig. 6B). Other immune‐related genes were intracellular immune signaling genes, suggesting that increased numbers of immune cells were contributing to the old lesion expression signature. Some ECM genes were also upregulated in the old tumors, indicating that both CAFs and immune cells were present, and inflammatory gene products known to be secreted by senescent fibroblasts (SASP markers) were noted (IL‐1β, IL‐7, Ccl3) (Fig. 6B).

### Increased inflammatory pathway activation in old mouse normal tissues and tumors

Validation of the microarray results by qPCR showed many inflammation‐associated genes (Ccl7, IL‐15ra, IL‐1β, and Cxcr6) as significantly upregulated in lung lesions from old *Kras* ^*G12D*^ mice (Fig. 6C–F). Of these, interleukin‐1β (IL‐1β) showed age‐dependent upregulation in control lungs and tumors from old mice, whereas tumor‐specific upregulation was observed for Ccl7, IL‐15ra, and Cxcr6 cytokines in old mice. In conjunction with increased cytokine levels detected in lung tumors from old mice, immunofluorescence analysis using αCD45 and αF4/80 showed increased accumulation of leukocytes and macrophages in close proximity to tumors from old mice (Fig. 6G,H, Fig. S7). Irrespective of age, most of the immune cells were present at the tumor periphery and not within the core of the tumors (Fig. S7).

The cytokine milieu plays a significant role in tumor promotion and progression. To further explore the age‐related inflammatory responses on associated lung tumors, broncho‐alveolar lung lavage fluid (BALF) from young and old *Kras* ^*G12D*^ mice 6 weeks after Cre adenovirus instillation was subjected to cytokine analysis. Luminex analysis showed that monocyte chemotactic protein, MCP‐1 (CCL2), was significantly elevated in BALF from old *Kras* ^*G12D*^ mice (Fig. S8A). Another key cytokine, IL‐6, that plays an important role in tumor progression, was higher in 5/12 old *Kras* ^*G12D*^ mice when compared to average levels of IL‐6 in young *Kras* ^*G12D*^ mice (*n* = 14). However, IL‐6 levels in BALF showed large variations and did not satisfy the test of significance (*P* = 0.1) when compared to BALF levels from young *Kras* ^*G12D*^ mice (Fig. S8B). The number of macrophages (*P* = 0.1), lymphocytes (*P* = 0.2), polymorphonuclear cells (*P* = 0.08), and total number of leukocytes (*P* = 0.08) in BALF from old *Kras* ^*G12D*^ mice tended to be elevated as compared to young *Kras* ^*G12D*^ mice (Fig. S8). Immune cell numbers in BALF from young and old controls were equivalent. The IL‐1β cytokine primarily secreted by activated macrophages is involved in inflammatory and immune response by inducing pro‐inflammatory genes such as IL‐6 and IL‐8. The serum level of IL‐1β was significantly higher in old *Kras* ^*G12D*^ mice than in young *Kras* ^*G12D*^ mice (Fig. S8D). The p38 MAPK signaling plays a key role in the production of several inflammatory regulators. Thus, lung sections from young and old *Kras* ^*G12D*^ mice were subjected to phospho‐p38 MAPK staining and lung tumors from old *Kras* ^*G12D*^ mice showed increased staining (Fig. S8E).

The activated inflammatory milieu as a function of age was further corroborated by experiments where differences were observed between young and old *LacZ* mice. Thus, serum levels for many cytokines such as IL‐6, IP‐10 (interferon‐induced protein‐10 also known as Cxcl10), MCP‐1 (monocyte chemoattractant protein‐1, also known as Ccl2), MIP‐2 (macrophage inflammatory protein‐2, also known as Cxcl2), and RANTES/Ccl5 were significantly elevated in old *LacZ* mice as compared to young *LacZ* mice (Fig. S9A). Similarly, lungs from old *LacZ* control mice showed higher levels of cytokines such as Cxcl2 and Cxcr2 by real‐time PCR analyses indicating elevated IL‐8 signaling (Fig. S9B‐C).

## Discussion

We sought to investigate the mechanisms by which aging tissues might influence cancer initiation and progression. Using a genetically engineered mouse model of inducible lung cancer, we examined the effects of activating the potent oncogene *Kras* ^*G12D*^ in young (3–5 months of age) and old mice (19–24 months of age). Lung‐specific activation of Kras^G12D^ resulted in hyperproliferation of the lung epithelial cells leading to hyperplasia, adenoma, and adenocarcinoma development in the lungs. Both long‐term survival and short‐term 6‐week studies showed accelerated rates of tumorigenesis with more aggressive tumors in 19‐ to 24‐month *Kras* ^*G12D*^ mice compared to their 3‐ to 5‐month counterparts. Significantly larger adenoma lesions were seen in old *Kras* ^*G12D*^ mice and in the timed 6‐week studies, only the old *Kras* ^*G12D*^ mice developed malignant adenocarcinomas (Figs 2A–D and 3A–C). In contrast to old *Kras* ^*G12D*^ mice, the young mice displayed larger numbers of lower grade broncho‐alveolar hyperplasias in the 6‐week study (Fig. 3G). One interpretation for this result is that preneoplastic lesions such as hyperplasias may arise at roughly similar rates in young and old mice in response to *Kras* ^*G12D*^ activation, but intrinsic and extrinsic processes in the young lung tissues are more effective in preventing progression of hyperplastic lesions to adenomas and adenocarcinomas.

Hyperproliferation induced by oncogene activation has been shown to be associated with replication stress and aberrant DNA strand breaks that activate the DNA damage response, forming a barrier to malignant progression (Halazonetis *et al*., 2008). With age, there is also accumulation of DNA lesions with unrepairable double‐strand breaks marked with γH2AX foci (Sedelnikova *et al*., 2004; Halazonetis *et al*., 2008; Schurman *et al*., 2012). Our data revealed reduced γH2AX staining in adenomas of old *Kras* ^*G12D*^ mice compared to adenomas in the young *Kras* ^*G12D*^ mice (Fig. 4C,D). Many of these lung adenomas in older mice had foci of neoplastic cells with cellular atypia as assessed by histopathological evaluation. In addition, DNA damage‐induced tumor suppressor Gadd45a transcripts were lower in healthy lungs and tumors from old mice (Fig. 4E). These data support the idea that premalignant cells in older tissues have a less robust DNA damage response than their younger counterparts.

The DNA damage response and the p53 tumor suppressor response are intertwined as damage response kinases activate p53 as well as DNA repair pathways (Sperka *et al*., 2012). The p53 damage response has been shown to decline with age in response to DNA damage in spleen and thymus of mice (Feng *et al*., 2007). In other studies, p53 activity was found to be upregulated in noncancerous tissues of aged mice (Edwards *et al*., 2007; Ugalde *et al*., 2011). We found a less efficient p53 tumor suppressor response, as measured by protein expression, in lung adenomas and hyperplasias from old *Kras* ^*G12D*^ mice. Analysis of other tumor suppressors shows that p19^Arf^ and p16^Ink4a^ are increased in expression in older normal mouse lung tissues, consistent with earlier findings that increased expression of these two genes is a general biomarker of normal tissue aging (Krishnamurthy *et al*., 2004). However, with induction of *Kras* ^*G12D*^, only young mouse adenomas show significant increases in p16^Ink4a^ and 19^Arf^ mRNA. Similarly, p21^Cip1^ mRNA levels are significantly upregulated in response to *Kras* ^*G12D*^ only in lung tumors from young *Kras* ^*G12D*^ mice but not in lung tumors from old *Kras* ^*G12D*^ mice relative to normal lung tissue (Fig. S4E). Such robust tumor suppressor upregulation in response to oncogenic stimulus specifically in young mice could represent an additional cancer progression barrier not available to older cells that already have high levels of expressed p16^Ink4A^, p19^Arf^, and p21^Cip1^ prior to oncogene activation.

In the discussion above, we focused on intrinsic factors specific to the developing lung cancer cell. However, extrinsic factors, particularly the tumor microenvironment, have been shown to play a major role in influencing the progression of a cancer lesion. A key component of the tumor microenvironment is the CAF that can become activated and reprogrammed by cancer cells to enhance synthesis of ECM structures and to secrete factors that attract immune cells of various types (Kalluri, 2016). Comparison of the global gene expression signatures of the young and old *Kras* ^*G12D*^ lung tumors shows that the young tumors preferentially express genes characteristic of activated CAFs and actively produce ECM components relative to their older counterparts. In contrast, the old tumors show preferential chemokine and cytokine expression as well as immune cell markers indicating a higher density of immune cells within the tumor microenvironment. Consistent with this, immunofluorescence analyses revealed elevated levels of immune cells in close proximity to the lung adenomas from old mice. While the increased cytokine expression in older tumors observed in Fig. 6A could be in part a result of larger tumor size (and thus more immune cells) in the older animals, our immunofluorescence scoring of immune cells in tumors was based on immune cell density (Fig. S7) and this density was significantly higher in older tumors independent of tumor size (Fig. 6G,H). Tumor‐derived chemotactic factors such as Ccl2, Ccl3, Ccl5, and Ccl7 are known to recruit macrophages to the growing tumor (Pollard, 2004; Sica *et al*., 2008). Additionally, enhanced inflammatory chemokine/cytokine expression has been shown to be a prominent phenotype in senescent fibroblasts that serve to directly and indirectly (in part through recruitment of tumor promoting immune cells) promote cancer cell growth and survival (Coppe *et al*., 2010; Lopez‐Otin *et al*., 2013). Other recent studies have emphasized the influence of contexts such as altered tissue microenvironment on age‐dependent incidence of cancers (Lasry & Ben‐Neriah, 2015; Rozhok & DeGregori, 2015).

Both aging and cancer being multifactorial pathophysiological states, it is not surprising that numerous factors work together and play an important role in the progression of cancer as a function of age. As illustrated in Fig. S10 (Supporting Information), our data point to an age‐dependent interplay between the cell intrinsic factors such as altered tumor suppressor responses and DNA damage response pathways with cell extrinsic factors exemplified by altered inflammatory and immune response pathways related to age‐associated tumor microenvironment changes. Other possible mechanistic factors not examined intensively in this study include age‐associated accumulation of somatic genetic and epigenetic alterations. Our demonstration that the putative tumor suppressor *Cdh13* is hypermethylated and downregulated in the lung tumors of old mice suggest that cell intrinsic epigenetic modifications may influence cancer progression in older animals, consistent with epigenetic studies in human cancers (Feinberg, 2007). The role of age‐associated acquisition of cooperating somatic cancer driver mutations was not examined in our model. The less than twofold increase in genomic somatic mutations within normal mouse tissues between the ages of 4 and 20 months (Vijg *et al*., 2005) argues against a largely somatic mutation‐driven mechanism for the age‐dependent tumorigenesis differences described here. Thus, nonmutational cell intrinsic and extrinsic mechanisms may play a larger role in age‐dependent cancer initiation and progression. Nevertheless, further experiments on human cancer systems and animal cancer models will be necessary to resolve these issues.

For methods, refer to Methods S1 (Supporting information) section.

## Author contributions

NP, RS, and LS performed the experiments. MG performed pathology and histopathology analyses. NP, LS, and LAD designed the experiments and analyzed the data. NP and LAD wrote the manuscript.

## Funding

This work was supported by R21 grant AG035365 from the National Institute on Aging. L.A.D.—NIH R21 AG035365; L.A.D.—Ellison Medical Foundation; N.P.—Ellison Medical Foundation and American Federation for Aging Research.

## Conflict of interest

None declared.

## Supporting information


**Fig. S1** Equivalent levels of LacZ activation in mouse lungs irrespective of age, six weeks post Cre adenovirus instillation.
**Fig. S2** Equivalent levels of LacZ activation in mouse lungs from young and old mice, two weeks post Cre adenovirus instillation.
**Fig. S3** Old *Kras* ^*G12D*^ mice developed more aggressive tumors than young *Kras* ^*G12D*^ mice.
**Fig. S4** Proliferation, DNA damage response and P19^Arf^, p16^Ink4a^, and p21^Cip1^ tumor suppressor responses to *Kras* ^*G12D*^ activation in young and old mice.
**Fig. S5** No significant age‐related changes are observed in global methylation in lungs whereas Cdh13 is downregulated and epigenetically regulated in old *Kras* ^*G12D*^ mice.
**Fig. S6** Lung tumors from young *Kras* ^*G12D*^ mice tend to show more robust staining for the fibroblast marker, alpha smooth muscle Actin (αSMA).
**Fig. S7** More immune cells cluster in close proximity to tumors from old mice.
**Fig. S8** Old *Kras* ^*G12D*^ mice show activated inflammatory pathways.
**Fig. S9** Control mice show significantly different cytokine and chemokine levels in serum and lung tissue as a function of age.
**Fig. S10** Model showing diverse cell intrinsic and cell extrinsic mechanisms that may influence tumor initiation and progression as a function of age.

**Table S1** Genes with significant enhanced expression in young lung adenomas relative to old adenomas.
**Table S2** GSEA canonical pathways significantly enriched in young adenomas relative to old adenomas.
**Table S3** Genes with significant increased expression in old lung adenomas relative to young adenomas.
**Table S4** GSEA canonical pathways significantly enriched in old adenomas relative to young adenomas.

**Methods S1** Experimental procedures.

**Data S1** Animal survival data.
