# Supplementary material for: Enhanced inflammation and attenuated tumor suppressor pathways are associated with oncogene‐induced lung tumors in aged mice
Source: Aging Cell. 2017 Oct 18;17(1):e12691. doi: 10.1111/acel.12691 (PMC5771401; doi:10.1111/acel.12691)

Supplementary Figure S1

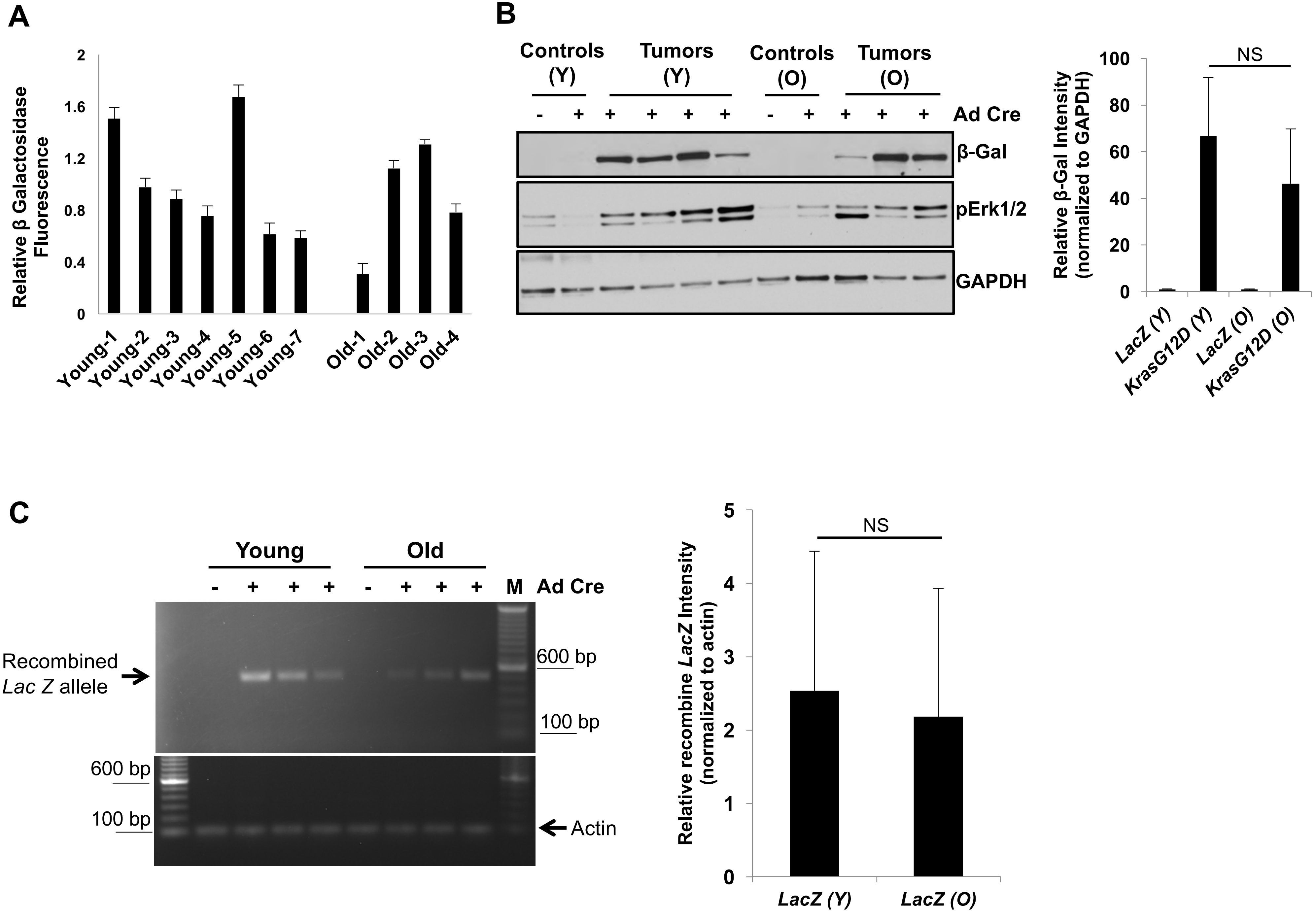

Supplementary Figure S2

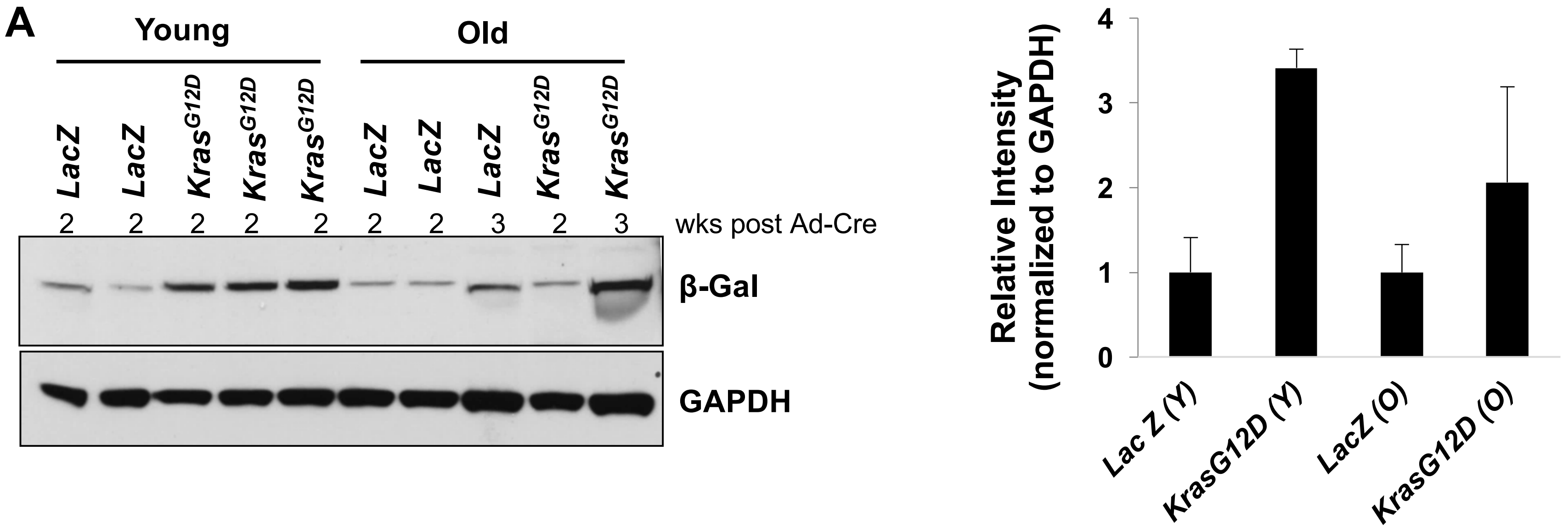

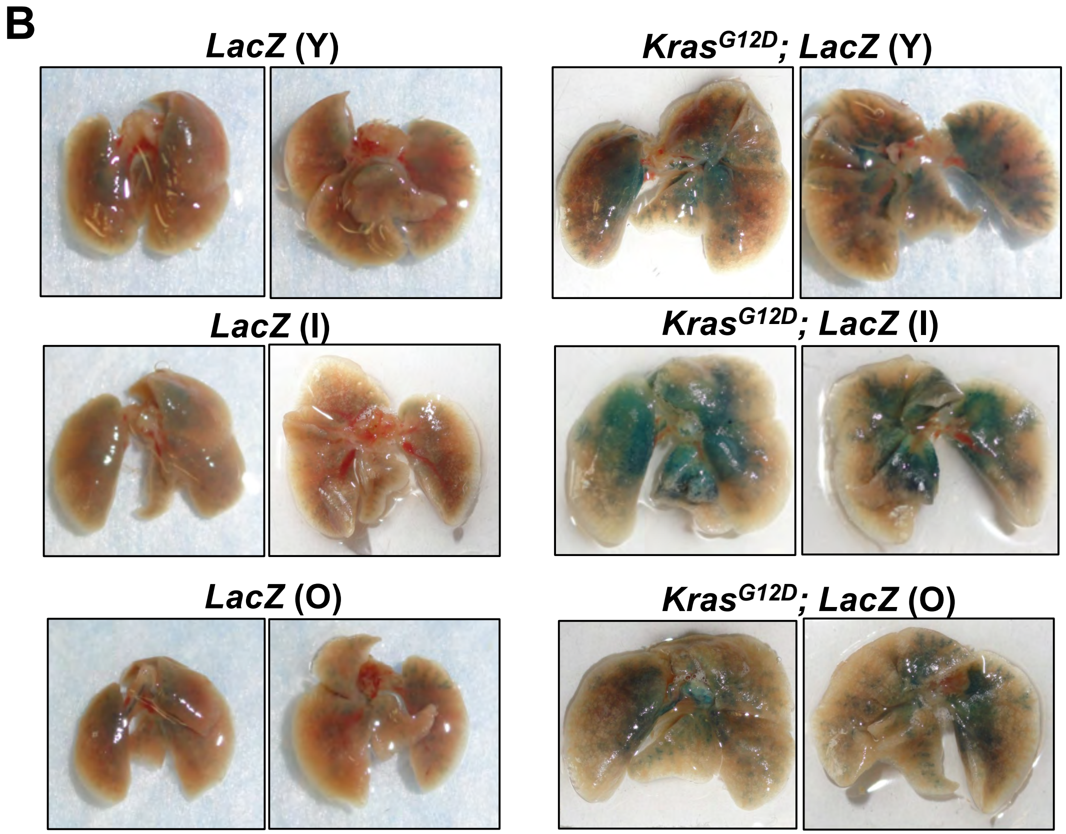

Supplementary Figure S3

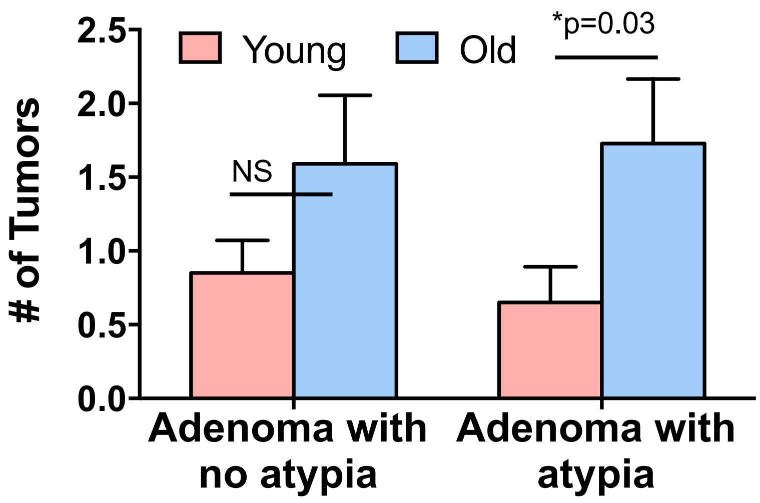

Supplementary Figure S4

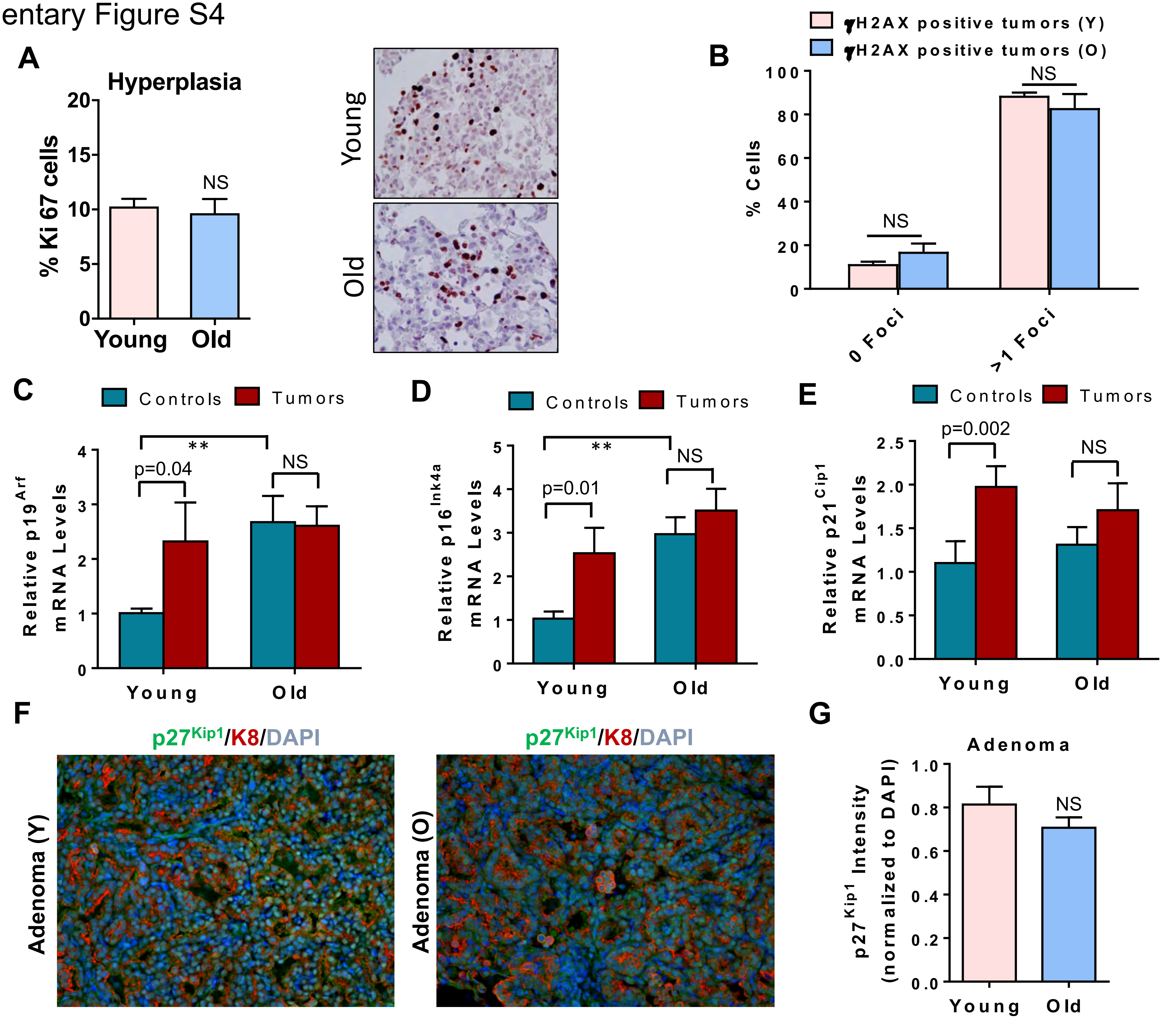

Supplementary Figure S5

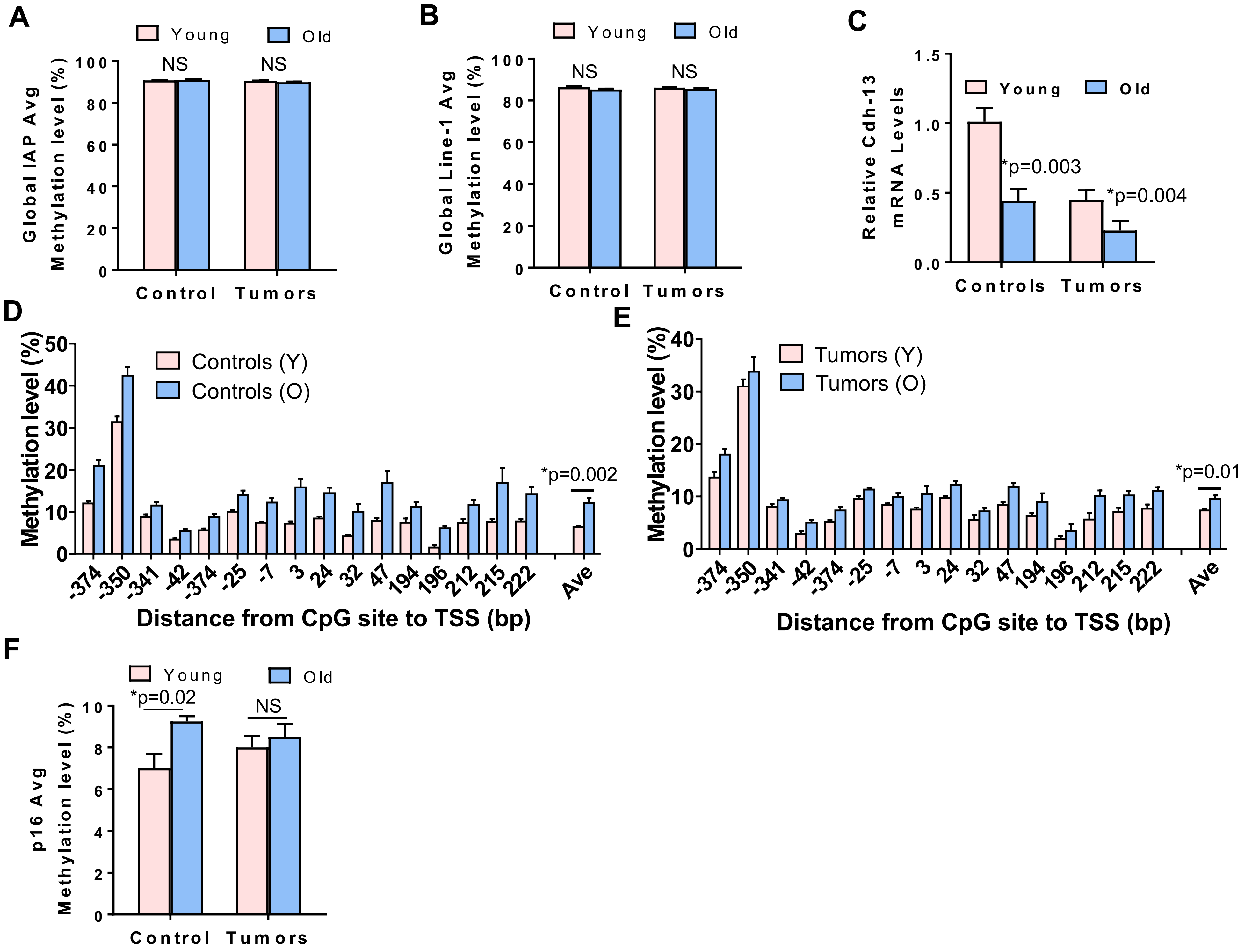

Supplementary Figure S6

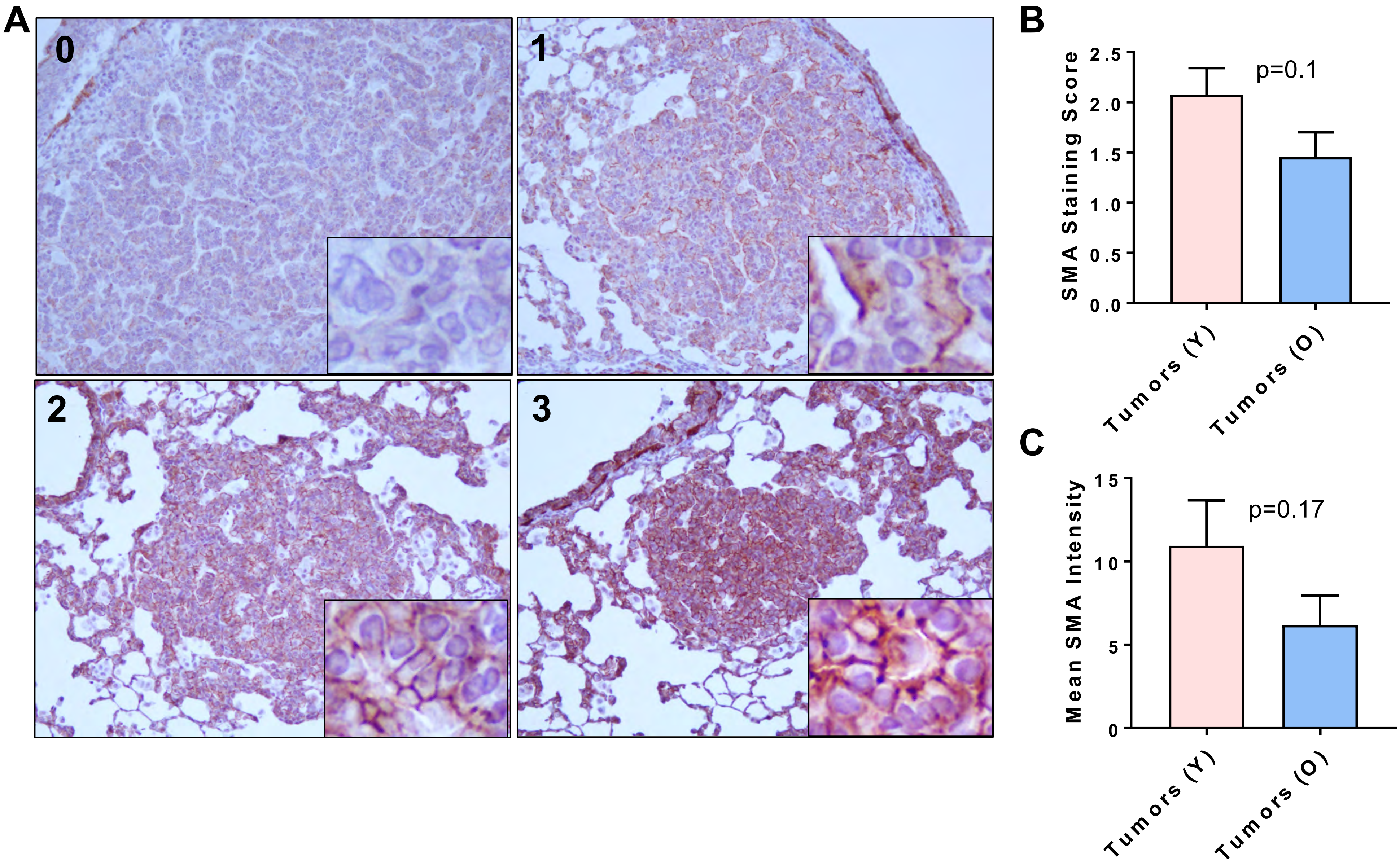

Supplementary Figure S7

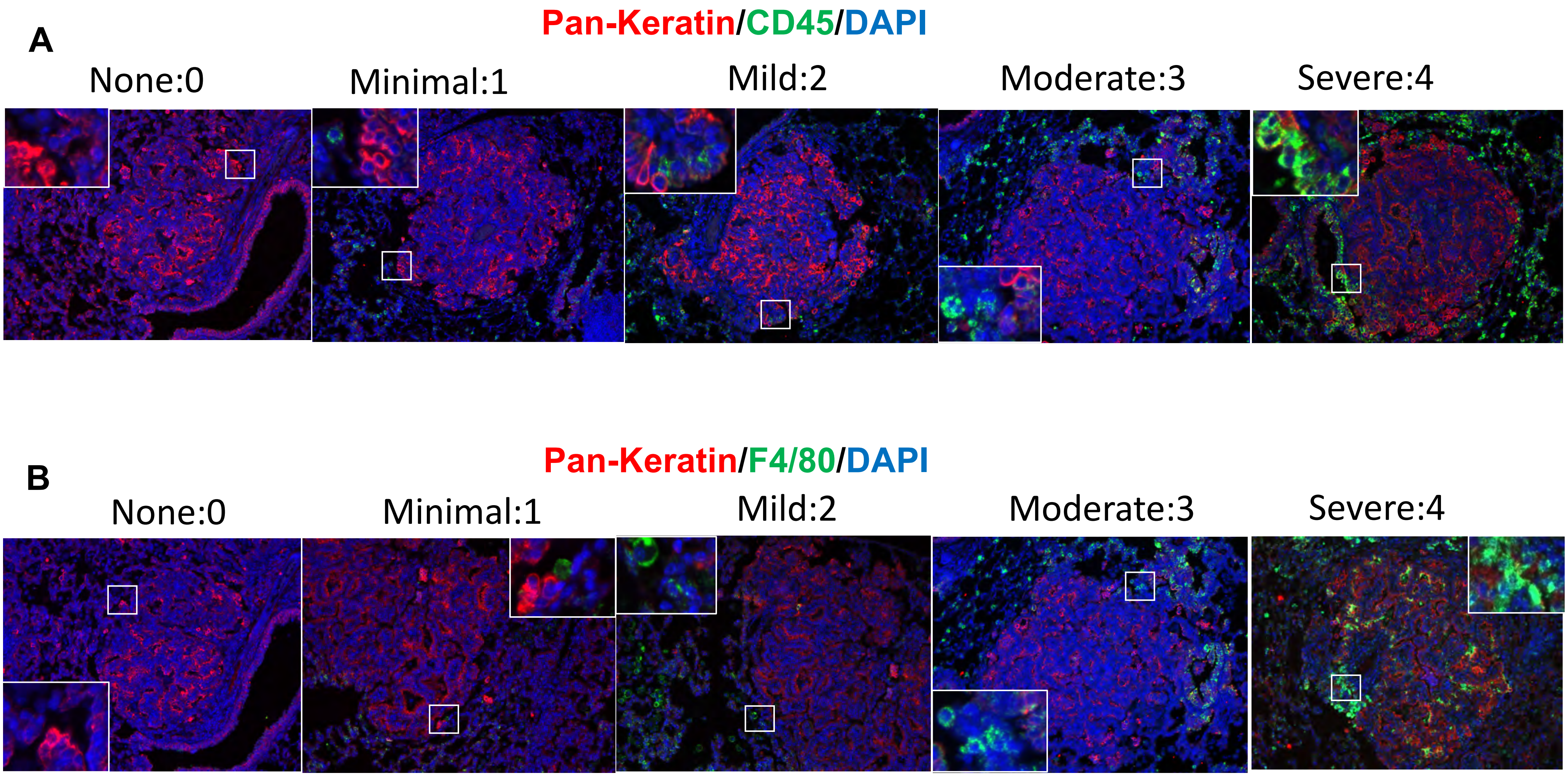

Supplementary Figure S8

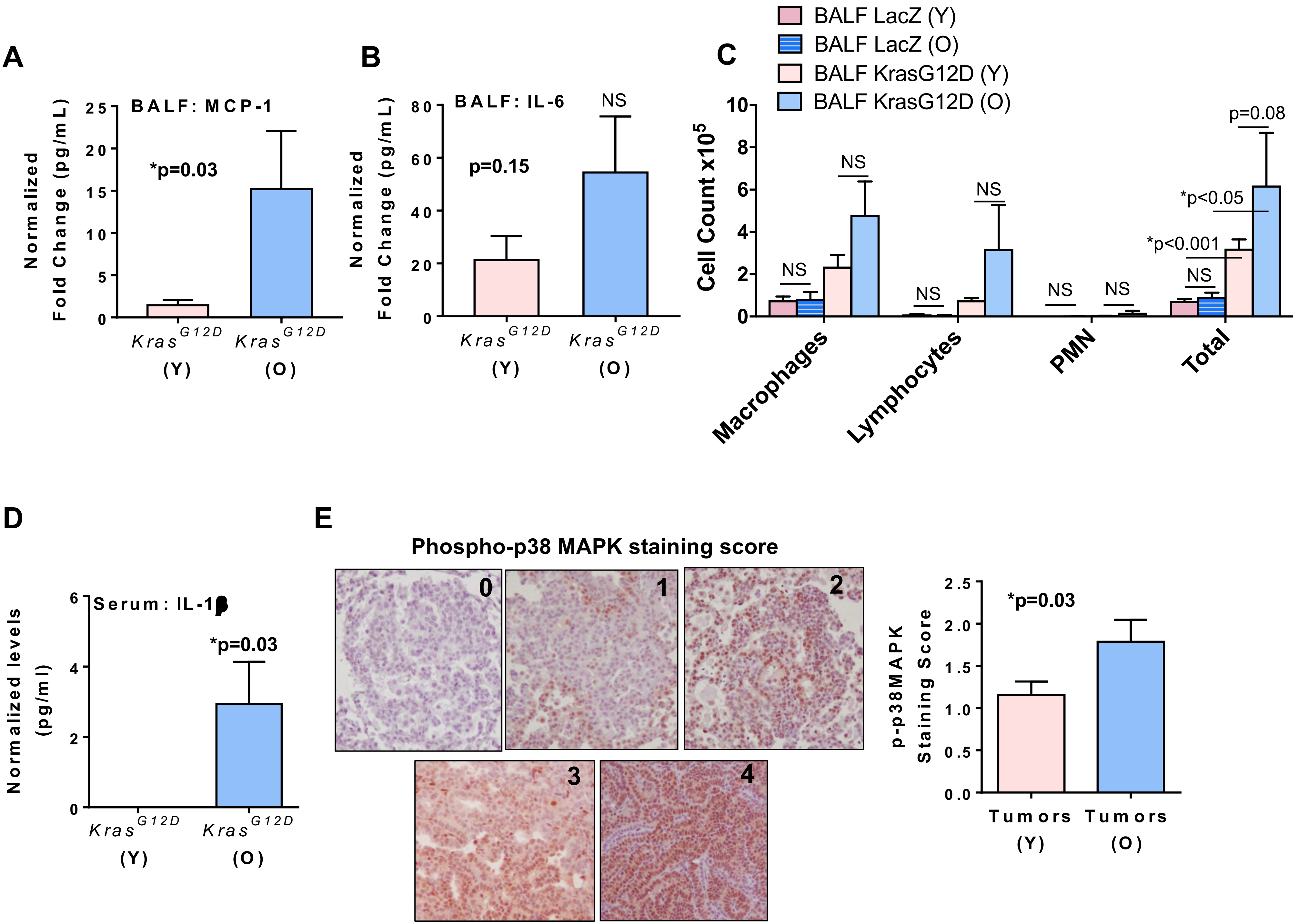

Supplementary Figure S9

A

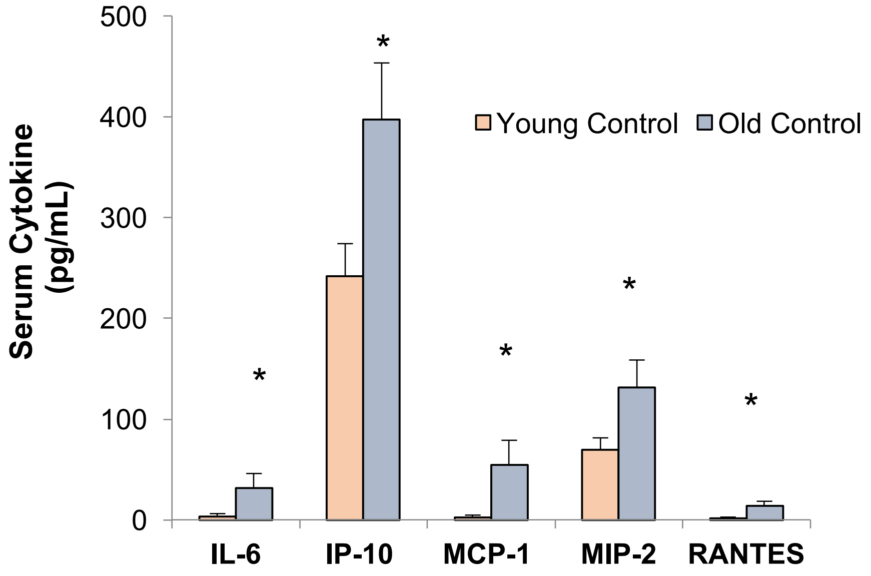

B

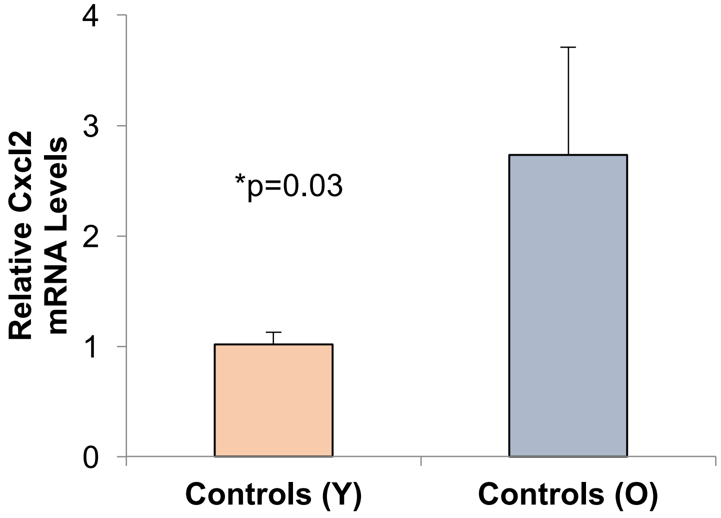

C

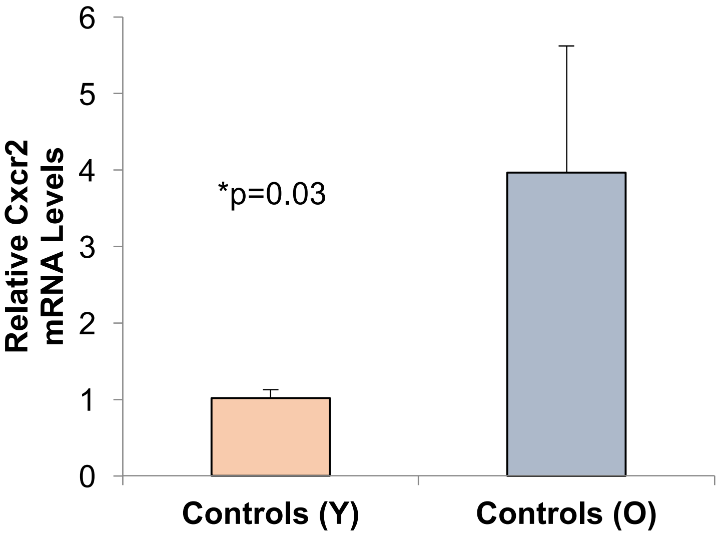

Supplementary Figure S10

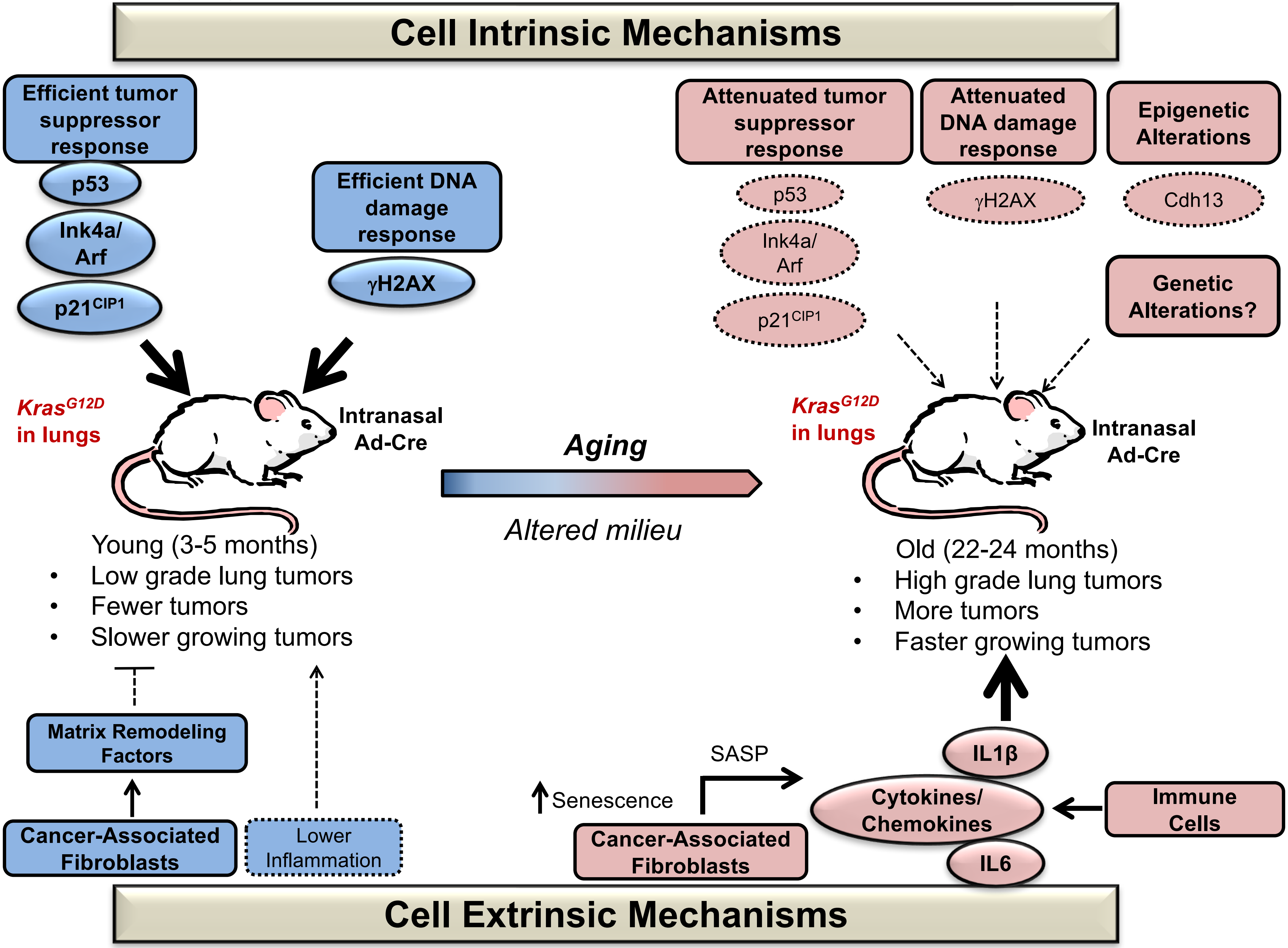

Supplement: Supplementary file 1 — Fig. S1 Equivalent levels of LacZ activation in mouse lungs irrespective of age, six weeks post Cre adenovirus instillation. Fig. S2 Equivalent levels of LacZ activation in mouse lungs from young and old mice, two weeks post Cre adenovirus instillation. Fig. S3 Old Kras G12D mice developed more aggressive tumors than young Kras G12D mice. Fig. S4 Proliferation, DNA damage response and P19Arf, p16Ink4a, and p21Cip1 tumor suppressor responses to Kras G12D activation in young and old mice. Fig. S5 No significant age‐related changes are observed in global methylation in lungs whereas Cdh13 is downregulated and epigenetically regulated in old Kras G12D mice. Fig. S6 Lung tumors from young Kras G12D mice tend to show more robust staining for the fibroblast marker, alpha smooth muscle Actin (αSMA). Fig. S7 More immune cells cluster in close proximity to tumors from old mice. Fig. S8 Old Kras G12D mice show activated inflammatory pathways. Fig. S9 Control mice show significantly different cytokine and chemokine levels in serum and lung tissue as a function of age. Fig. S10 Model showing diverse cell intrinsic and cell extrinsic mechanisms that may influence tumor initiation and progression as a function of age. [file ACEL-17-na-s001.pdf]
