# Supplementary material for: Enhanced inflammation and attenuated tumor suppressor pathways are associated with oncogene‐induced lung tumors in aged mice
Source: Aging Cell. 2017 Oct 18;17(1):e12691. doi: 10.1111/acel.12691 (PMC5771401; doi:10.1111/acel.12691)
Supplement: Supplementary file 3 — Methods S1 Experimental procedures. [file ACEL-17-na-s003.docx]

**Experimental Procedures**

**Mice**

B6.129 background *LSL-Kras^G12D^* mice (donated by Dr. Tyler Jacks, Massachusetts Institute of Technology, Cambridge, MA, USA) and backcrossed with C57BL/6 mice for 9-10 generations were obtained from the National Cancer Institute Mouse Repository. These mice were crossed into C57BL/6 background *Rosa26-LSL LacZ* (R26R) mice developed by Dr. Phillippe Soriano, Mount Sinai School of Medicine and obtained from Jacksons Labs (Bar Harbor, ME, USA). All mice were bred and maintained in a specific pathogen-free animal facility at Baylor College of Medicine. All research with mice was conducted in compliance with the Baylor Animal Protocol Committee (Baylor College of Medicine Animal Protocol AN-336) and AAALAC recommendations as published in The Guide for the Care and Use of Laboratory Animals (NRC1996).

**Allele genotyping**

Genomic DNA was prepared from 5 mm tail tips or 50 mg tissue as described (Parikh *et al.* 2012). Genotyping for the *LSL-Kras^G12D^* allele was performed using the Fwd primer, 5’ AGC TAG CCA CCA TGG CTT GAG TAA GTC TGC 3’ and Rev primer 5’ CCT TTA CAA GCG CAC GCA GAC TGT AGA 3’ giving a 550 bp PCR product for the LSL allele. Genotyping for the *LacZ* allele in homozygous R26R mice was performed using the Fwd 5’ GCG AAG AGT TTG TCC TCA ACC 3’ and Rev 5’ GGA GCG GGA GAA ATG GAT ATG 3’ primers yielding a 340 bp product for the mutant.

**PCR detection of LSL recombination**

Genomic DNA from control *LacZ* lungs were subjected to standard PCR using Fwd Primer 5’ AAA GTC GCT CTG AGT TGT TAT 3’ and Rev Primer 5’ GTT GTA AAA CGA CGG GAT CC 3’ yielding a product of ~500bp for the *LoxP* recombined allele. The non-recombined *LacZ* allele was too big to be amplified with the PCR conditions used. β Actin Fwd Primer 5’ CACTGTCGAGTCGCGTCC 3’ and β Actin Rev Primer 5’ TCATCCATGGCGAACTGGTG 3’ were used as control.

**Intranasal instillation of Cre adenovirus and *Kras^G12D^* allele induction**

Cre adenovirus Ad5-CMV-Cre was obtained from the Vector Development Laboratory at Baylor College of Medicine. For the efficient delivery and transduction of Cre adenovirus to lungs, AdCre:CaPi co-precipitates were formed by mixing 1.25x10^6^ PFU of adenovirus with 10 mM calcium chloride as per protocol (DuPage *et al.* 2009). Briefly 1.25x10^6^ PFU of Cre adenovirus was mixed with MEM and calcium chloride. The mixture was incubated for 20 minutes at room temperature and was used for intranasal instillation within 1 hr of preparation. For the intranasal administration, mice were anesthetized in an airtight chamber purged with a 2% isoflurane in oxygen vapor mixture for 5 min to achieve deep general anesthesia. To facilitate virus delivery to lungs, anesthetized mice were held in an upright manner with gentle pressure locking the lower jawline while slowly adding Cre adenovirus: CaPi co-precipitates (total volume of 50 μl) droplets to the nostrils with a pipette. Mice were allowed to gradually inhale the droplets before returning to the cage for recovery. The tips in contact with adenovirus were disinfected with 50% bleach. All procedures were carried out in a bio-safety hood. For survival analysis, 1.25x10^6^ PFU of Cre adenovirus was instilled in young (3-5m) and old (~19m) *Kras^G12D^* mice. Mice at the moribund stage were humanely sacrificed as per guidelines and lungs harvested for further analysis. In the survival study, old age mice that died due to causes other than lung tumor (mostly lymphoma), were omitted from the survival study. For the molecular analyses, a short-term study was employed whereby 1.25x10^6^ PFU of Cre adenovirus was instilled in young (3-5m) and old (~22-24m) *Kras^G12D^* mice. Mice were humanely sacrificed 6 weeks post Cre adenovirus as per guidelines and lungs harvested for further analysis. The ages referred to in the text are the ages of mice at which Cre adenovirus was instilled.

**Histopathological analyses of lung tumors**

H&E stained lung sections were analyzed by the pathologist (MG) blinded to the identity of lung sections. As per published literature (Jackson *et al.* 2001; DuPage *et al.* 2009), lung bronchioloalveolar hyperplasia is characterized by hypertrophy and proliferation of alveolar pneumocytes and terminal bronchiolar cells leading to disruption of the single cell lining of the alveolar sacs and thickening of alveolar walls. Lung bronchioloalveolar adenoma is a benign tumor characterized as a well-demarcated solid tissue mass composed of hyperplastic monotonous alveolar pneumocytes with bland nuclei, which typically arises from a single clone of cells. Lung bronchioloalveolar adenocarcinoma is a malignant tumor characterized either by presence of neoplastic pneumocytes with highly pleomorphic nuclei, marked anisokaryosis, increased number of mitotic cells, poorly defined borders and peripheral invasion, or by tumors greater than 3-4 mm in diameter.

**Morphometric analysis of lung tumors**

Serial hematoxylin and eosin (H&E) stained sections of all lung lobes were scanned with Aperio AT2® slide scanner (Leica Biosystems) at 20X magnification. Microscopic digital images of lungs were examined by an experienced veterinary pathologist in a blinded manner. The lung tumors and other lesions were measured with Aperio ImageScope software, and the number and area of each tumor and lung lobe were analyzed.

**Tissue lysate preparation and Western blot analysis**

Freshly excised tissue samples were harvested, snap frozen, and stored at -80°C until use. Western blotting was performed as described before (Parikh *et al.* 2012). Blots were incubated with antibodies for pErk1/2 (Cat # 4370) from Cell Signaling Technology (Danvers, MA, USA), β-galactosidase (Cat# Z378A, Promega Corporation, Madison, WI, USA) and GAPDH (Cat #MAB374) from MilliporeSigma (Billerica, MA, USA).

**Histologic Analysis and Immunohistochemistry**

For histological analysis, lungs and tumors were perfused and fixed with 10% neutral buffered formalin. Fixed tissues were processed, embedded in paraffin blocks, and cut in 5 µm thick sections that were mounted on glass slides and stained with H&E. H&E stained sections of all lung lobes were examined histopathologically and the lung tumors were analyzed and classified in a blinded manner by veterinary pathologist M.G. Immunohistochemistry (IHC) was performed as described previously (Parikh *et al.* 2012) using the Vectastain ABC kit Rabbit (Cat #6101, Vector Labs, Burlingame, CA, USA) and Vector M.O.M. Immunodetection Kit (Vector Labs, Cat #2202) as per manufacturer’s instructions. The sections were incubated with the following primary antibodies (4°C overnight): anti αSMA (Cat # ab32575, abcam, Cambridge, MA), anti phospho p38MAPK (Cell Signaling Technology, Cat #4511), anti γH2AX (MilliporeSigma, Cat # 05-636), anti-Keratin 8 (TROMA-I, deposited by P. Brulet and R. Kemler at Developmental Studies Hybridoma Bank, University of Iowa, Iowa City, IA, USA) and anti Ki67 (Vector Labs, Cat #VP-RM04). Bright field images were captured using an inverted microscope (BX50, Olympus, Center Valley, PA, USA) equipped with Olympus DP11 camera. The percent of Ki-67 positive cells was quantified by Inform software analysis of lung tumors imaged using 10X objective. Percentage for each tumor was calculated and used to obtain the mean score for the young and old groups. For large tumors, multiple micrographs were quantitated and average percent was used to calculate the mean score for the group. The αSMA staining was quantitated by Image J 1.51n software using constant threshold and default method. Mean gray value obtained from tumor images was plotted as mean intensity. For large tumors, multiple micrographs were quantitated and average intensity for the tumor was plotted.

**Immunofluorescence**

The immunofluorescence was developed as described (Parikh *et al.* 2012). Briefly paraffin sections were blocked at room temperature for 1 hr in a TSA kit blocking buffer (Perkin Elmer, Waltham, MA) and incubated overnight at 4°C or room temperature with the following antibodies: anti β-galactosidase (Promega Corporation), anti-p27^Kip1^ (Santa Cruz Biotechnology, Dallas, TX, USA), anti-p53 (P53-CM5P-L, Leica Biosystems, New Castle Upon Tyne, United Kingdom), anti F4/80 (Cat # ab6640, abcam, Cambridge, MA), anti CD45 (Cat # 550539, BD Biosciences, San Jose, CA), pan keratin (Cat # ab6401, abcam) and anti-Keratin 8. The Keratin 8 and pan keratin was probed using Alexa Fluor conjugated secondary antibodies (Thermo Fisher Scientific). Fluorescent images were captured at 20X with a Nikon TE-2000 Epi-Fluorescence Microscope inverted microscope and images processed using Image J and NIS elements software. For nuclear p53 and p27^Kip1^ immunofluorescence quantitation analysis, merge of p53/p27^Kip1^ and DAPI were run through MATLAB to obtain nuclear overlap images of p53/p27^Kip1^ using constant threshold filters across each experiment. Average nuclear p53/p27^Kip1^ percent intensity in lung lesions was normalized to percent DAPI intensity in the same field and plotted. For percent β galactosidase positive cells, immunostained images were analyzed by MATLAB using a constant threshold for all the images. Five-six randomly captured image fields per lung section were averaged and plotted for each animal.

**Lung lavage and cytokine analysis**

One ml PBS was instilled into the mouse lungs and the lavage fluid was slowly collected back in a syringe. 50-100µl of lavage fluid was subjected to cytospin and stained using Hema3 stain. Total cell count was determined using a hemocytometer. Remaining lavage fluid was spun down, and supernatant stored in -80°C for cytokine analysis. Cytokine detection assays using luminex bead technology were performed at the Antibody-based Proteomics Core at Baylor College of Medicine.

**X-Gal Staining**

Lungs harvested from mice were fixed with 4% PFA for 2 hrs at 4°C followed by intra-tracheal perfusion of X-Gal solution (1 mg/ml diluted in tissue stain base solution, MilliporeSigma). Lungs were then incubated overnight at 37°C in X-Gal solution.

**Microarray Analysis**

Lung tumors isolated by visual examination at the time of necropsy were subjected to RNA extraction using TRIzol (Thermo Fisher Scientific) and Norgen RNA Cleanup Kit (Thorold, ON, Canada). Three hundred nanograms of total RNA were amplified and purified using Illumina TotalPrep RNA Amplification Kit (Thermo Fisher Scientific, Cat# IL1791) following kit instructions. Briefly, first strand cDNA was synthesized by incubating RNA with T7 oligo(dT) primer and reverse transcriptase mix at 42ºC for 2 hrs. RNase H and DNA polymerase master mix were immediately added into the reaction mix following reverse transcription and were incubated for 2 hrs at 16ºC to synthesize second strand cDNA. RNA, primers, enzymes and salts that would inhibit in vitro transcription were removed through cDNA filter cartridges in the amplification kit. In vitro transcription was performed and biotinylated cRNA was synthesized by 14-hr amplification with dNTP mix containing biotin-dUTP and T7 RNA polymerase. Amplified cRNA was subsequently purified and concentrations measured by NanoDrop ND-1000 Spectrophotometer (Thermo Fisher Scientific). An aliquot of 750 ng of amplified products were loaded onto Illumina Sentrix Beadchip Array Mouse Ref8_v2 arrays, hybridized at 58ºC in an Illumina Hybridization Oven (Illumina, Cat# 198361) for 17 hrs, washed and incubated with straptavidin-Cy3 to detect biotin-labeled cRNA on the arrays. Arrays were dried and scanned with a BeadArray Reader (Illumina, San Diego, CA, USA). Data were analyzed using GenomeStudio software (Illumina). Clustering and pathway analysis were performed with GenomeStudio and Ingenuity Pathway Analysis (Ingenuity Systems, Inc., Germantown, MD, USA) software, respectively. For quality control and pre-processing, raw signals of all the build-in controls were checked as quality control for the performance of the arrays. Sample-independent controls were used to check hybridization (control molecules at low, medium and high concentrations) and signal generation (background, noise, biotin labeling and hybridization at high and low stringency). Housekeeping genes were used as sample-dependent controls. Background was subtracted and arrays were normalized using *quantile*. The reproducibility of biological or technical replicates was checked through comparisons among individual samples. Outliers were removed if necessary. The remaining samples were grouped and the average signal intensities of samples within the group were used for differential expression analysis.

**Quantitative real-time PCR**

Total RNA extracted from mouse tissues was reverse transcribed and real-time PCR was performed in the StepOnePlus Real time PCR System (Thermo Fisher Scientific) using Power SYBR® Green Master Mix (Thermo Fisher Scientific). The following primers were designed using Primer-BLAST software: p19^Arf^ Fwd 5’ TCACTGTGAGGATTCAGCGCGC 3’ and p19^Arf^ Rev 5’ GCCCATCATCATCACCTGGTCC 3’; p16^Ink4a^ Fwd 5’ CTCGAGGAGAGCCATCTGGAGCAG and p16^Ink4a^ Rev 5’GAGCTGCTACGTGAACGTTGCCC 3’, p21^Cip1^ Fwd 5’ CGGTGTCAGAGTCTAGGGGA 3’ and p21^Cip1^ Rev 5’ AGGATTGGACATGGTGCCTG 3’; Cdh13 Fwd 5’ CAGACAGTCCCTGATAAAGTC 3’ and Cdh13 Rev 5’ TGGGCAGGTTGTAGTTTGC 3’; Ccl7 Fwd 5’ CCAATGCATCCACATGCTGC 3’ and Ccl7 Rev 5’ TGTCTTGAAGATAACAGCTTCCCA 3’; Il1β Fwd 5’ GCCACCTTTTGACAGTGATGAG 3’ and Il-1β Rev 5’ GACAGCCCAGGTCAAAGGTT 3’; Cxcr6 Fwd 5’ AGAGAACCAGAGGCAGACCT 3’ and Cxcr6 Rev 5’ TCCATGGCATCAGAAGAGAAGC 3’; Il15ra Fwd 5’ GGTCACTGCTGGGGACAAT 3’ and Il-15ra Rev 5’ GTGGTGCCCGGCGTC 3’; Gadd45ra Fwd 5’ TGGTGACGAACCCACATTCA 3’; and Gadd45ra Rev 5’ CGGGAGATTAATCACGGGCA 3’; β Actin Fwd 5’ CACTGTCGAGTCGCGTCC 3’ and β Actin Rev 5’ TCATCCATGGCGAACTGGTG 3’. Relative fold change was calculated with respect to young control lungs and normalized to expression of β Actin using the ΔΔCt method.

**DNA methylation analysis**

Quantitative bisulfite-pyrosequencing for DNA methylation analyses was performed as previously described (Shen *et al.* 2007). Following primer sets and sequences were used for *Cdh13* promoter CpG:

1. PCR Primer Fwd 5’ GGTGTAATTTTAGTTTTTAGGGAAAGT and Rev 5’ [Btn] ACTAATAACCAAAACCAATAACTTTACAA; Sequencing Primer 1: AAAAAAAAAAAAAAAAAAAAGTTAA and Sequencing Primer 2: GAGGTGTTAGTTTTTATTTG; Sequence 1 analyzed: YGTGTYGTTTTTTTGTTYGGAGGGGGTTTTTTTTGYGAGGTGTTYGTTTT and Sequence 2 analyzed: TTATGTAAAAYGAGGGAGYGTTATGAAGGAATTYGTTTTG
2. PCR Primer Fwd 5’ AGGTTGGTTTTTAAGGAAAATATGTTTAG and Rev 5’ [Btn] CTAACTACCACTCACATTCCCTACCTAA; Sequencing Primer: GGAAAATATGTTTAGTGTAG; Sequence analyzed: TYGYGTGTGTGAATGTAAAYGTYGTTAGGYGTTTTTTTTAGT
3. PCR Primer Fwd 5’ GTGATTTGTGGTGATGGTAGAA and Rev 5’ [Btn] ACCCTCTATACCCTAAAAATCTTAC; Sequencing Primer: GGTGATGGTAGAAAAATATTAAAT; Sequence analyzed: ATTATYGGTGGTTGAGTTATTTTGGGGGTYGATGGGGGYGGGGGGGGGGTGAGGGGGAGAGG

The p16^Ink4a^ promoter methylation and global DNA methylation analysis (based on Line1 and IAP) were performed as described previously (Yu *et al.* 2014). For each assay, set-up included positive controls (*SssI*-treated genomic DNA) and negative controls (whole genome amplified genomic DNA), mixing experiments to rule out bias, and repeated experiments to assess reproducibility. Annealing temperatures were optimized to overcome PCR bias as previously reported (Shen *et al.* 2007).

**References**

DuPage M, Dooley AL, Jacks T (2009). Conditional mouse lung cancer models using adenoviral or lentiviral delivery of Cre recombinase. *Nature protocols*. **4**, 1064-1072.

Jackson EL, Willis N, Mercer K, Bronson RT, Crowley D, Montoya R, Jacks T, Tuveson DA (2001). Analysis of lung tumor initiation and progression using conditional expression of oncogenic K-ras. *Genes & development*. **15**, 3243-3248.

Parikh N, Shuck RL, Nguyen TA, Herron A, Donehower LA (2012). Mouse tissues that undergo neoplastic progression after K-Ras activation are distinguished by nuclear translocation of phospho-Erk1/2 and robust tumor suppressor responses. *Molecular cancer research : MCR*. **10**, 845-855.

Shen L, Guo Y, Chen X, Ahmed S, Issa JP (2007). Optimizing annealing temperature overcomes bias in bisulfite PCR methylation analysis. *BioTechniques*. **42**, 48, 50, 52 passim.

Yu DH, Waterland RA, Zhang P, Schady D, Chen MH, Guan Y, Gadkari M, Shen L (2014). Targeted p16(Ink4a) epimutation causes tumorigenesis and reduces survival in mice. *The Journal of clinical investigation*. **124**, 3708-3712.
