# Supplementary material for: Enhanced inflammation and attenuated tumor suppressor pathways are associated with oncogene‐induced lung tumors in aged mice
Source: Aging Cell. 2017 Oct 18;17(1):e12691. doi: 10.1111/acel.12691 (PMC5771401; doi:10.1111/acel.12691)
Supplement: Supplementary file 5 [file ACEL-17-na-s005.docx]

**Supplementary Figure Legends**

**Figure S1. Equivalent levels of LacZ activation in mouse lungs irrespective of age, six weeks post Cre adenovirus instillation.** (A) **%** β-galactosidase positive cells as measured by immunofluorescence using a beta-galactosidase-specific antibody from individual young or old *LacZ* control mice are shown in the bar graph. (B) Lungs or lung lesions identified by gross morphology at the time of dissection and harvested 6 weeks post Cre adenovirus instillation were assessed for activation of Kras signaling (pErk1/2) and expression of β-galactosidase in young (Y) and old (O) mice as shown. GAPDH served as a loading control. Right panel shows quantitation of β-galactosidase expression normalized to GAPDH levels (C) Genomic DNA from *LacZ* control lungs was PCR amplified using forward primer upstream of the LSL cassette and reverse primer binding at the 5’ end of *Lac Z* allele. The deletion of LSL cassette following Cre Adenovirus instillation (+) allows the detection of a 550 bp PCR product whereas in the absence of Cre-mediated recombination (-), the predicted PCR product is too large to be amplified under the employed PCR conditions. PCR with actin primers served as a control for equivalent amounts of genomic DNA used as input. Right panel shows quantitation of recombined *LacZ* allele PCR product from Ad Cre instilled samples normalized to actin levels. NS: not significant.

**Figure S2.** **Equivalent levels of LacZ activation in mouse lungs from young and old mice,** two weeks post Cre adenovirus instillation (A) Lungs from *LacZ* and *Kras^G12D^* young and old mice harvested 2 weeks post Cre adenovirus instillation were assessed for expression of β-galactosidase as shown. GAPDH served as a loading control. Right panel shows quantitation of mean β-galactosidase protein expression in *LacZ* (Y), *LacZ* (O), *Kras^G12D^* (Y), and *Kras^G12D^* (O) lungs relative to GAPDH protein expression (B) Comparable X-Gal staining was observed in lungs harvested 2 weeks after Cre adenovirus instillation in young (Y, 3-4 m), intermediate (I, 8-9 m) and old (O, 18-19 m) mice.

**Figure S3.** **Old *Kras^G12D^* mice developed more aggressive tumors than young *Kras^G12D^* mice.** Significantly higher numbers of more aggressive adenoma with atypia were observed in old *Kras^G12D^* mice. Average numbers of adenoma with no atypia and adenoma with atypia lesions observed per median lung section per mouse are plotted (young: 3-5 months, n=20; old: 22-24 months, n=19)

**Figure S4. Proliferation, DNA damage response and P19^Arf^, p16^Ink4a^, and p21^Cip1^ tumor suppressor responses to *Kras^G12D^* activation in young and old mice.** (A) Quantitation of Ki67 positive cells in lung hyperplasia from young (16 lung hyperplasia from 5 mice) and old (15 lung hyperplasia from 5 mice). *Kras^G12D^* mice shows equivalent proliferation index in early lung lesions. Right panels: Images with Ki67 immunostaining in lung hyperplasias of an old *Kras^G12D^* mouse (O, 22-24 months) and a young *Kras^G12D^* mouse (Y, 3-5 months) six weeks post Cre adenovirus. (B). Lung tumors graded positive for γH2AX foci from young (Y) and old (O) mice were further assessed for % cells with no foci and >1 foci present in nuclei. Data from 5 young (8 lesions) and 5 old (15 lesions) mice is plotted. (C) Quantitative RT-PCR for p19^Arf^ (C) and p16^Ink4a^ (D) mRNA showed increased expression of both messages in young *Kras^G12D^*-activated lesions relative to young normal lung tissue, whereas *Kras^G12D^* activation in old tissues show little effect on p16^INK4a^ and p19^Arf^ RNA expression, which are already high in normal tissues. Young control lungs (3-5 m, n=3), old control lungs (22-24 m, n=3), young (3-5 m, n=5) and old (22-24 m, n=5) *Kras^G12D^* lesions were examined. (E) p21^Cip1^ mRNA expression measured by quantitative RT-PCR showed a significant robust response to *Kras^G12D^* activation in young lung tissues, but not in old lung tissues. (F) No significant change in p27 immunostaining (green) is observed in lung adenomas from young and old *Kras^G12D^* mice. Blue represents DAPI stained nuclei and red represents keratin 8 (K8) stained epithelial cells. Representative images from young (Y) and old (O) mice are shown. (G) Quantitation of p27 intensity overlapping with nuclei and normalized to percent DAPI intensity in adenomas from young (9 lesions from 5 mice) and old (10 lesions from 5 mice) mice is plotted. NS: not significant, **p<0.0001.

**Figure S5. No significant age-related changes are observed in global methylation in lungs whereas Cdh13 is downregulated and epigenetically regulated in old *Kras^G12D^* mice.** (A and B) DNA methylation analysis of IAP (A) and Line-1 (B) promoter CpG islands in young and old control lungs (n=5) and lung tumors (n=6) show no significant differences (NS). The percentage methylation of individual CpG sites was measured by quantitative bisulfite-pyrosequencing and average % methylation was plotted. (C) Cdh13 mRNA levels are downregulated in lung tumors from old *Kras^G12D^* mice as assessed by qPCR (n=5 for young and old *Kras^G12D^* mice). (D and E) Increased DNA methylation of the *Cdh13* promoter CpG island are observed in control lungs (D, n=5) and lung tumors from old (O) *Kras^G12D^* mice (C, n=6) as compared to control lungs (E, n=5) and lung tumors from young (Y) mice (C, n=6). The percentage methylation of individual CpG sites between -374 to +222 bp relative to transcription start site (TSS) was measured by quantitative bisulfite-pyrosequencing. (F) The *Cdkn2a* (*p16^Ink4a^*) promoter exhibits moderately increased methylation in old lung tissue relative to young tissue, whereas lung tumors show no significant age-related differences in *Cdkn2a* (*p16^Ink4a^*) promoter methylation.

**Figure S6. Lung tumors from young *Kras^G12D^* mice tend to show more robust staining for the fibroblast marker, alpha smooth muscle Actin (αSMA). (A)** Representative images of lung adenoma stained for αSMA (reddish brown) and counterstained with hematoxylin (purplish blue) along with various scores for αSMA staining are shown. Inset shows enlarged images of tumor cells (purplish blue) surrounded by fibroblasts cells marked with αSMA (reddish brown) staining. Lung tumors were given a score of 0-3 based on visual assessment of the extent and intensity of αSMA staining. (B and C) Average αSMA staining score obtained from visual assessment (B) and average mean intensity of αSMA staining obtained from Image J based quantitation (C) shows a trend for more pronounced staining in tumors from young mice (13 lung adenoma tumors from 4 young (Y) *Kras^G12D^* mice and 11 lung adenoma tumors from 4 old (O) *Kras^G12D^* mice were examined).

**Figure S7. More immune cells cluster in close proximity to tumors from old mice.** (A and B) Lung adenomas from young and old mice were immunostained for leukocytes with αCD45 (A, green) and macrophages **with** αF4/80 antibody (B, green). Blue represents DAPI stained nuclei and red represents pan keratin antibody marked epithelial cells in lung tumors. Representative images with 0-4 score based on estimates of immune cells clustering around tumor periphery (within 100 µm distance) or within the tumor core are shown. Inset shows enlarged images of cells with CD45 (A) or F4/80 (B) staining. The following convention was used by the pathologist to assign score for immunostained lung tumors. Grade 0 = no histologic detection (or normal tissue), Grade 1 = minimal, rare, infrequent, or barely noticeable feature (affects 1-10% of the tissue), Grade 2 = mild, slight, infrequent, sporadic, noticeable but not prominent feature (affects 11-20% of tissue), Grade 3 = moderate, frequent, typical, common, prominent feature (affects 21-40% of tissue), Grade 4 = marked, extensive, numerous, severe, overwhelming feature (affects 41-100% of tissue).

**Figure S8.** **Old *Kras^G12D^* mice show activated inflammatory pathways.** (A) Broncho-alveolar lung lavage fluid (BALF) levels of MCP-1 are elevated in old *Kras^G12D^* mice. BALF was extracted six weeks after Cre intranasal instillation of young (3-5 m) and old *Kras^G12D^* mice (22-24 m) and subjected to cytokine analysis. Data for MCP-1 in *Kras^G12D^* mice normalized to *LacZ* controls is plotted. Old *LacZ* and old *Kras^G12D^* mice: n=12, Young LacZ mice: n=13, Young *Kras^G12D^* mice: n=15. (B) Same as panel A except that data for IL-6 is plotted. (C) Relatively elevated levels of BALF cell population is observed in old *Kras^G12D^* mice. No age-related difference is observed in BALF cell population from healthy control lungs. PMN: Polymorphonuclear leukocytes. n=8-13 (Old *Kras^G12D^*); n=10-16 (young *Kras^G12D^*); n=5 (old and young *LacZ*). (D) Increased level of IL-1β is detected in serum from old *Kras^G12D^* mice. IL-1β level from young (n=7) and old (n=6) *Kras^G12D^* mice normalized to *LacZ* controls is plotted. (E) Increased p-p38 MAPK immunostaining is observed in lung tumor sections from old *Kras^G12D^* mice six weeks after Cre adenovirus instillation. Tumors were given a score of 0-4 based on visual assessment of the extent and intensity of p-p38 MAPK staining. Representative images of lung lesions (severe focal hyperplasia, adenoma and adenocarcinoma) stained for p-p38MAPK (reddish brown) and counterstained with hematoxylin along with various scores for p-p38MAPK staining are shown. Right panel shows average p-p38 MAPK staining score obtained from young (Y) and old (O) *Kras^G12D^* mice (19 lesions from 4 young mice and 14 lesions from 7 old mice)

**Figure S9.** **Control mice show significantly different cytokine and chemokine levels in serum and lung tissue as a function of age.** (A) Increased cytokines levels are seen in serum obtained from old control (22-24 months, n=7) mice. Young (3-5 months, n=8) and old control mice were instilled with Cre adenovirus or left untreated. Six weeks later serum these mice was analyzed for cytokines levels *p<0.05. (B-C) Quantitative RT-PCR analysis for Cxcl2 (C, n=3 for young and old mice) and Cxcr2 (D, n=3 for young and old mice) shows upregulation in lungs of old (22-24 months) mice. Lungs were harvested from young (3-5 months) and old (22-24 months) *LacZ* mice six weeks after Cre adenovirus instillation.

**Figure S10. Model showing diverse cell intrinsic and cell extrinsic mechanisms that may influence tumor initiation and progression as a function of age.** Young tissues are shown to have robust cell intrinsic DNA repair and tumor suppressor response mechanisms to suppress or minimize initial oncogenic stimuli such as *Kras* mutation. In the aged tissue environment, however, in addition to attenuation of the cell intrinsic DNA damage and tumor suppressor responses, a cell extrinsic chronic inflammatory environment resulting from changes in the tumor microenvironment associated with increased numbers of senescent cancer-associated fibroblasts. These senescent cells exhibit a senescence-associated secretory phenotype (SASP) that attracts immune cells that in turn produce factors that may further promote neoplastic progression. The above cell intrinsic and extrinsic processes may complement intrinsic somatic acquisition of genetic and epigenetic changes that influence the cancer phenotype.
